# Phylogeography of the ant *Myrmica rubra* and its inquiline social parasite

**DOI:** 10.1002/ece3.6

**Published:** 2011-09

**Authors:** Jenni Leppänen, Kari Vepsäläinen, Riitta Savolainen

**Affiliations:** Department of BiosciencesP.O. Box 65, 00014University of HelsinkiFinland

**Keywords:** Hymenoptera, inquilinism, Pleistocene glaciations, postglacial recolonization, social parasitism, speciation

## Abstract

Widely distributed Palearctic insects are ideal to study phylogeographic patterns owing to their high potential to survive in many Pleistocene refugia and—after the glaciation—to recolonize vast, continuous areas. Nevertheless, such species have received little phylogeographic attention. Here, we investigated the Pleistocene refugia and subsequent postglacial colonization of the common, abundant, and widely distributed ant *Myrmica rubra* over most of its Palearctic area, using mitochondrial DNA (mtDNA). The western and eastern populations of *M. rubra* belonged predominantly to separate haplogroups, which formed a broad secondary contact zone in Central Europe. The distribution of genetic diversity and haplogroups implied that *M. rubra* survived the last glaciation in multiple refugia located over an extensive area from Iberia in the west to Siberia in the east, and colonized its present areas of distribution along several routes. The matrilineal genetic structure of *M. rubra* was probably formed during the last glaciation and subsequent postglacial expansion. Additionally, because *M. rubra* has two queen morphs, the obligately socially parasitic microgyne and its macrogyne host, we tested the suggested speciation of the parasite. Locally, the parasite and host usually belonged to the same haplogroup but differed in haplotype frequencies. This indicates that genetic differentiation between the morphs is a universal pattern and thus incipient, sympatric speciation of the parasite from its host is possible. If speciation is taking place, however, it is not yet visible as lineage sorting of the mtDNA between the morphs.

## Introduction

The genetic constitution of species living today is partly a result of demographic processes during the Pleistocene climatic cycles (Avise 2000; Hewitt 2004). In the Pleistocene era, distribution and abundance of populations were altered by changing environmental conditions during each glacial and interglacial period. At the end of the last glaciation, during the last glacial maximum (LGM) ca. 20,000 years ago, a large part of Eurasia was covered by ice (Svendsen et al. 2004), and populations of temperate species were predominantly restricted to areas referred to as glacial refugia (Hewitt 1999). After the ice sheet started to retreat, many of the populations spread out from the refugia and recolonized areas previously covered by ice (Hewitt 1999). During postglacial recolonization, where divergent genetic lineages of species met, secondary contact zones with high genetic diversity originated (Hewitt 2004). Nevertheless, today most species are phylogeographically structured, displaying regionally restricted genetic lineages (Avise 2009).

Traditionally, the main refugia of the European temperate fauna and flora have been placed in southern Europe—in the Iberian, Apennine, and Balkan peninsulas (Taberlet et al. 1998; Hewitt 1999; Stewart et al. 2010). Indications of refugia have, however, been found for some species in Turkey, the Caspian–Caucasus region (Taberlet et al. 1998; Hewitt 1999; Stewart et al. 2010), and further east in the southern parts of the Ural Mountains (Bilton et al. 1998; Polyakov et al. 2001; Goropashnaya et al. 2004b, 2007) and in southern Siberia (Grichuk 1992; Polyakov et al. 2001; Goropashnaya et al. 2004b). In Europe, also more northern refugia may have existed, for example, in the northern Carpathians, the Pannonian Plains, and southern France (Pfenninger and Posada 2002; Willis and van Andel 2004; Sommer and Nadachowski 2006; Ursenbacher et al. 2006; Stewart et al. 2010). Consequently, the importance of the southern European peninsulas as main refugia and sources of postglacial colonization has recently been questioned in several species (Stewart et al. 2010). Anyhow, even though some species have survived the last glaciation in non-Mediterranean refugia, their importance for temperate species in general remains unclear (Stewart et al. 2010).

Lately, several researchers have pointed out that—to understand better phylogeographic patterns in Europe—studies on many additional taxa are needed (Avise 2009; Hickerson et al. 2010; Stewart et al. 2010). We share this viewpoint for the following reasons. First, phylogeographic studies of Palaearctic species have often covered only part of the range of the species, and second, the sampling may have been too scarce to reveal the phylogeographic pattern of the species (Avise 2000; Hewitt 2004; Gómez and Lunt 2007). Thus, conclusions on refugia and postglacial colonization routes of the species may be biased in their emphasis on the most intensively studied European refugia or they may be unreliable owing to insufficient sampling of populations.

To expand the comprehension of the phylogeography of the western Palaearctic fauna and flora, we suggest that suitable study organisms should: (1) be common and abundant, (2) have extensive, continuous temperate–boreal Palaearctic distributions, and (3) be relatively cold-tolerant. Taxa such as these have had a high potential to survive in many refugia and, after the glaciation, to recolonize vast areas in the continuous zone suitable for the species. Consequently, the potential to survive in many refugia is likely to decrease the extinction risk of the species during the glaciation, and to increase the genetic differences among the populations during the long glacial isolation—both factors that will increase the genetic diversity of the present-day species (Hewitt 1996, 1999; Avise 2009). Thus, a mixture of different postglacial lineages may expand the ecological tolerance of the species, which will contribute to their commonness and abundance.

Species that meet the above criteria (1–3) include many Palearctic insect taxa, but these have received phylogeographically little attention (Wahlberg and Saccheri 2007). For example, several ant genera (Hymenoptera: Formicidae) include species that are suitable phylogeographic study organisms, but of them only species of *Formica* have been studied in the context of Palearctic refugia (Goropashnaya et al. 2004a, b, 2007). These studies did not, however, find phylogeographical structure within species, perhaps owing to too scarce sampling.

Our selected study organism, the ant *Myrmica rubra* (Linnaeus), is a perennial, cold-tolerant species (Kipyatkov 2001) with a Palaearctic range from the British Isles to eastern Siberia and from Fennoscandia to the northern Iberian and Apennine Peninsulas and the Balkans, and to the Altai Mountains in southern Siberia (Czechowski et al. 2002; Radchenko and Elmes 2010). *Myrmica rubra* is common and abundant throughout most of its distributional area owing to its wide ecological tolerance: it occurs in a diverse array of habitats, particularly in meadows, open broad-leaved and mixed forests, and gardens (Czechowski et al. 2002). Since *M. rubra* is abundant and morphologically stable over its wide distribution, Radchenko and Elmes (2010) suggested that *M. rubra* is an ideal species to study phylogeographic history. While acknowledging the lack and need of its molecular phylogeography, they proposed that *M. rubra* survived the last ice age in Middle Asia, the Balkans, or southeast Europe.

**Figure 1:**
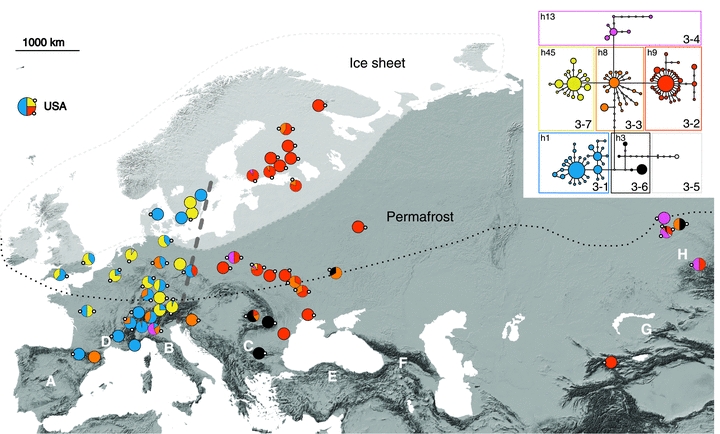
Haplogroup distribution map with haplotype network of Figure 3. Dashed line represents west–east division of haplogroups, white area approximates limit of ice sheet (Svendsen et al. 2004) and dotted line that of permafrost during the last glacial maximum (Renssen and Vandenberghe 2003; Saito et al. 2009). Potential refugia of *M. rubra* shown with white letters: A = Iberian Peninsula, B = the Apennine Peninsula, C = the Balkans, D = southern France, E = Turkey, F = the Caucasus, G = Middle Asia, H = Siberia. Pie charts show proportions of third-level haplogroups in their respective colors of haplotype network; size of pie chart is not proportional to sample size. A single pie chart represents one location, except for following combined neighbor-population sets separated by semicolon (population numbers in Table 1): 2, 39; 6–8; 9–10; 11, 16, 21–24; 14–15; 18, 20; 28, 29; 35, 38; 40–46; 50, 52; 53, 54, 56; 55, 58; 59, 65; 62, 63; 69, 72; 70, 74; 77–79; 81–82; 83, 87; 88–90, 92–93; 94–95. Local occurrence of common, central haplotype and its haplogroup identity is shown by small white circle at periphery of pie chart; identity number of each central haplotype is given in haplotype network insert, at upper left corner of its third-level haplogroup box.

Here, we tested the above single-refugium hypothesis of Radchenko and Elmes (2010), and the following alternative multiple-refugia hypothesis. On the basis of the extensive distribution and wide ecological tolerance of *M. rubra*, we suggest that the species survived the last ice age in several refugia—scattered over an expansive area from west to east—and recolonized its present areas of distribution along several routes. To localize these potential refugia, we used the data on climatic (Ganguly et al. 2009) and vegetation zones (Allen et al. 2010) within the present range of *M. rubra* and its main habitats (Czechowski et al. 2002). We compared the present climate and vegetation zones with those during the LGM using reconstructions of climate (Renssen and Vandenberghe 2003; Van Huissteden et al. 2003; Saito et al. 2009; Strandberg et al. 2011) and vegetation data (Frenzel 1992; Grichuk 1992; Tarasov et al. 2000; Willis and van Andel 2004; Allen et al. 2010) to deduce the locations of suitable refugia for *M. rubra* (marked with white letters in Fig. 1). We tested the existence of these putative refugia situated in the southern European peninsulas (A–C) and in more northern (D) and eastern regions (E–H).

Our extensive sampling also enabled us to study the mitochondrial DNA (mtDNA) differentiation of the peculiar life forms of *M. rubra*. Namely, it has two size classes of queens, large macrogynes and small microgynes (Elmes 1973). The microgynes are inquilines, that is, functionally workerless, intraspecific obligate social parasites of the macrogynes (Elmes 1978). The two queen morphs produce only queens of their own kind, which indicates that the size dimorphism is genetically regulated (Elmes 1976). Based on the size differences between the morphs, the microgyne morph was described as a separate species, *M. microrubra* (Seifert 1993). It was, however, owing to limited genetic differentiation between the morphs, synonymized with *M. rubra*—moreover, incipient speciation of the microgyne parasite was categorically denied (Steiner et al. 2006). Nevertheless, in a microsatellite study of two populations in southern Finland, the *M. rubra* macrogyne and its microgynous parasite differed genetically (Vepsäläinen et al. 2009), which suggests that the parasite may be speciating from its host (Savolainen and Vepsäläinen 2003; Seifert 2007, 2010; Vepsäläinen et al. 2009). Such a population-level genetic differentiation between the morphs—if shown to be of a general pattern, found wherever the two morphs coexist—would imply incipient speciation of the inquiline. Since the inquiline is, through its entire life, fully dependent on the host morph, if speciation takes place, it cannot be anything but sympatric. Consequently, here we appraised two competing hypotheses, the no-speciation versus incipient-speciation hypothesis, on a large geographical scale.

## Materials and Methods

### Data

We collected 241 nest samples of *Myrmica rubra* from 60 localities in the Palearctic, including many sites within the putative refugia or their adjacent areas. We selected nests located at least 3 m apart and collected from each nest all castes of the host and parasite (if available), and preserved them in absolute ethanol. We also obtained through our colleagues 103 nest samples from 28 additional localities in the Palearctic, and four nest samples from two localities in Maine and Massachusetts, where *M. rubra* is an invasive species (Groden et al. 2005; Wetterer and Radchenko 2010). In addition, we used part of the *M. rubra* samples of Vepsäläinen et al. (2009, Table S1). Finally, we used from GenBank 47 cytochrome c oxidase I (COI) and 21 cytochrome b (Cyt b) sequences (Table S1). These came from 31 nests in six localities (from the locality FRmo we also obtained our own data). Thus, our data consisted of 419 specimens from 379 nests in 95 localities (169 host queens, 202 host workers, 48 parasite queens; Tables S1 and S2). The 48 parasites came from 47 nests in 24 localities, from 22 nests we obtained the host queen, and from 15 nests we used the host worker.

### DNA sequencing

From each nest, we used one host and one parasite queen, if available, for sequencing. We extracted DNA from legs with the salt extraction method (Aljanabi and Martinez 1997), and sequenced partial mitochondrial COI and Cyt b genes of each individual. For COI, we used the universal primers LCO1490 and HCO2198 (Folmer et al. 1994) and for Cyt b, the ant-specific primers CB7 and tRs (Tay et al. 1997). From the samples of Vepsäläinen et al. (2009), we only sequenced Cyt b since COI was available in GenBank. We amplified and sequenced the gene fragments using the protocol of Jansen et al. (2009; for Cyt b we used 46°C for annealing temperature). We compiled, edited, and aligned the sequences with Sequencher 4.5 (Gene Codes). The length of the COI sequence was 565 bp and of Cyt b 863 bp, in total 1428 bp. Of the queens (*n* = 202) for which we extracted DNA from, we measured the mesosoma length using a Leica MZ9_5_ stereomicroscope with 10× oculars and a Planapo 1.6× objective (Leica Microsystems, Heerbrugg, Switzerland), to identify them as host and parasite morphs as specified by Seifert (1993).

### Analyses

To test the two hypotheses about refugia and postglacial colonization, we assessed the geographic distribution of genetic diversity. To locate areas of high genetic variation, which are most likely to be either refugial areas or secondary contact zones of genetic lineages (Hewitt 1996), we studied genetic diversity within and among populations. We calculated genetic diversity for all populations, treating local host and parasite samples separately. First, we analyzed sequence polymorphism and defined the haplotypes in the software DnaSP v5 (Librado and Rozas 2009). Then we calculated haplotype diversity (*h*) and nucleotide diversity (π) for each host and parasite population with the program Arlequin 3.11 (Excoffier et al. 2005). Haplotype diversity (equivalent to expected heterozygosity in diploid loci) uses observed haplotype frequencies, and is the probability that two randomly chosen haplotypes differ in a population; nucleotide diversity (mean number of nucleotide differences between all haplotype pairs in the sample) is the probability that two randomly chosen homologous nucleotide sites differ in a population (Nei 1987).

To survey the genetic diversity geographically, we applied two approaches to the Eurasian host populations. First, using the Mantel test (Mantel 1967) and 1000 replicates in the program Alleles In Space (AIS, Miller 2005), we tested correlation between logarithmically transformed genetic and geographic distances of individuals, and thus the effect of isolation by distance. Second, we used AIS to reconstruct a genetic landscape for a graphical representation of areal genetic diversity (Miller et al. 2006). The analysis is based on a three-dimensional surface plot where *x*- and *y*-axes correspond to geographic coordinates and *z*-axis (height) to genetic distance. We used the Delaunay triangulation-based connectivity network to connect neighboring sampling locations to each other. Between each neighboring pair of locations, the program calculates genetic distance that results in a certain height in the topography. The peaks indicate large genetic distances between neighboring locations and, thus, high areal genetic diversity. To avoid possible effect of isolation by distance on the results, we used the residual genetic distances. For the total area covered (2204 km^2^), we specified an 89 × 24 grid and 0.5 as a distance-weighting parameter.

We studied genetic differentiation (1) within *M. rubra* hosts, (2) within *M. rubra* parasites, and (3) between *M. rubra* hosts and their parasites with Φ -statistics (Excoffier et al. 1992). In Arlequin, we first calculated pairwise Φ_ST_-values based on pairwise differences and frequencies of haplotypes between all Eurasian host populations, between all parasite populations, and between local host and parasite populations with 1000 permutations. Next, we tested our hypothesis on genetic differentiation of *M. rubra* and its parasite. We applied the Analysis of Molecular Variance (AMOVA, Excoffier et al. 1992) to partition the genetic diversity hierarchically among and within local host and parasite populations. We used the AMOVA with two alternative highest level groupings: queen morphs and populations. We compared the groups for (1) pairwise differences and frequencies of haplotypes, and (2) only haplotype frequencies, with significance tests based on 20,000 permutations. We then applied DnaSP to obtain the average number of pairwise nucleotide differences between local host and parasite populations.

To compare genetic connections of geographic areas and to infer locations of glacial refugia and postglacial recolonization routes, we reconstructed a haplotype network using statistical parsimony (Templeton et al. 1992). Haplotype networks use information on inferred mutational steps between the haplotypes and group closely related haplotypes together. We reconstructed the haplotype network of all sequences with the program TCS 1.21 (Clement et al. 2000) with 95% connection limit. We resolved ambiguous connections (loops) between the haplotypes according to Templeton et al. (1995) and then hierarchically divided the haplotypes into nested haplogroups with the program aNeCa (Panchal 2007; Panchal and Beaumont 2007), using the rules of Templeton et al. (1987). According to these rules, tip and interior haplotype(s) were clustered into first-level haplogroups. These haplogroups were then further clustered into higher level groups step by step until only a single haplogroup remained.

We used Fu's *F_S_* test of selective neutrality, which is based on the infinite-site model (Fu 1997), and a mismatch distribution analysis (Schneider and Excoffier 1999) to assess whether *M. rubra* haplogroups have experienced recent expansions. Recent population expansion results in an excess number of alleles and negative Fu's *F_S_* value (Fu 1997). The mismatch distribution shows the observed number of nucleotide differences between pairs of sequences (Rogers and Harpending 1992). The distribution is usually multimodal in populations at demographic equilibrium and unimodal in ones that have gone through a recent population expansion. Both analyses are based on the assumption that the data come from one panmictic population (Rogers 1995; Fu 1997). We included in the analyses only the Eurasian host individuals and performed the analyses for all third-level haplogroups except 3–5 that had only one haplotype. We did both tests in Arlequin with 1000 replicates. We fitted the observed mismatch distribution of each group against the stepwise demographic expansion model (Rogers and Harpending 1992; Rogers 1995) and used the *P*-value test of Schneider and Excoffier (1999). For those groups that fitted the model, we estimated tau (τ) with 95% confidence intervals (CI). Tau measures time on a mutational scale; in mismatch analyses, τ corresponds to the peak of the mismatch distribution and can be used to estimate the time since expansion as pairwise nucleotide differences (Rogers and Harpending 1992). The time in years since expansion (*t*) can be estimated applying the equation *t* = τ/2*u,* where *u* is the mutation rate of the sequences calculated from *u* = *m_t_*µ, where *m_t_* is the length of sequence (1428 bp) and µ the mutation rate per nucleotide year.

We estimated the time since divergence (*t*) of haplogroups as follows. The climate began to warm approximately 15 ka BP (where ka = kilo years, Hubberten et al. 2004) and the Scandinavian ice sheet disappeared approximately 10 ka BP (Wohlfarth et al. 2008), when the earliest colonizers, such as birches, were already present in most of Europe (Palme et al. 2003). As young birch forests are prime habitats of *M. rubra* within its main range, we presumed that population expansion of *M. rubra* followed the advance of birch forests soon after the LGM approximately 10–15 ka BP. Assuming that the tip haplotypes, found only in the areas previously covered by glacial ice, have been formed postglacially, then the expansion of the haplogroups occurred after the LGM. Thus we used tau (τ) values of three haplogroups that showed the strongest signs of recent expansion and included many tip haplotypes that were sampled only in the areas previously covered by glacial ice: 3-1 (Italian haplotypes excluded), 3-2 and 3-7, to estimate minimum and maximum mutation rates. If the expansion (*t*) of these haplogroups occurred 10–15 ka BP, then according to the equation *t* = τ/2*u*, the mutation rate would be 4–5% Ma^−1^.

We estimated divergence times of the haplogroups by using the net distance approach in MEGA v.4 (Tamura et al. 2007), which corrects for heterogeneity of sequences within groups (Nei and Li 1979). For this purpose—when the genetic distances are calculated from closely related sequences—complex distance measures are unnecessary (Nei and Kumar 2000), and thus we used the Jukes–Cantor substitution model and calculated standard errors for the estimates with 10,000 bootstrap replicates. We estimated the time since divergence (*t*) from equation *d = 2rt*, where *d* is the number of substitutions and *r* the nucleotide substitution rate per site per year. Substitution rates of insect mitochondrial genes vary substantially (Hewitt 1996; Dowton et al. 2009; Ferreira et al. 2009; Papadopoulou et al. 2010; Pons et al. 2010), but those of the genus *Myrmica* are not known. Thus, we used both the general substitution rate of insects, 1.15% Ma^−1^ (Brower 1994), and the two substitution rates determined by us above from the expansion estimate, 4% Ma^−1^ and 5% Ma^−1^. We calculated 95% CI for the time since divergence as ±1.96 standard error of the net distances.

## Results

### Genetic diversity and population structure

The COI gene fragment (565 bp) had 34 polymorphic sites and 40 haplotypes, and the Cyt b gene fragment (863 bp) had 77 polymorphic sites and 70 haplotypes. Neither fragment included stop codons or ambiguous regions. The combined gene fragments (1428 bp) included 101 haplotypes (Tables 1, S1, and S2). The sequences differed on average at 5.9 nucleotides (min–max = 0–26), and the maximum observed divergence between two sequences was 1.8%. Of the 111 variable sites, 31 were singletons and 80 parsimony-informative. In the pooled data of host and parasite individuals, haplotype diversity (*h*) was 0.95 and nucleotide diversity (π) 0.0041.

**Table 1:** Population (*n* = 95) details; country, population code (population numbers and capital letters referring to country, lowercase letters to area within country), population coordinates in decimal degrees (N = latitude, E = longitude), numbers of individuals sampled (hosts and parasites), haplotypes and haplogroups for each population with numbers of individuals (*n*)

		**Coordinates**		**Individuals**			
					
**Country**	**Population**	**N**	**E**	**Host**	**Parasite**	**Haplotypes (*n*)**	**Haplogroups (*n*)**
Andorra	1. AN	42.50	1.56	12	–	47 (12)	3–3 (12)
Austria	2. AUac	47.55	11.69	5	–	1 (1), 45 (3), 50 (1)	3–1 (1), 3–7 (4)
Belgium	3. BEbr	50.84	4.37	15	–	1 (3), 30 (1), 45 (10), 48 (1)	3–1 (3), 3–7 (11), 3–5 (1)
Bulgaria	4. BLvi	42.53	23.37	12	–	3 (11), 4 (1)	3–6 (12)
Denmark	5. DKmo	56.20	10.52	1	–	1 (1)	3–1 (1)
England	6. ENdoki	50.62	–2.12	9	–	1 (8), 82 (1)	3–1 (9)
	7. ENdore	50.60	–2.05	7	–	81 (7)	3–7 (7)
	8. ENdose	50.64	–1.95	1	1	1 (2)	3–1 (2)
	9. ENnoea	52.60	1.26	2	–	80 (2)	3–7 (2)
	10. ENnoun	52.63	1.24	3	–	45 (1), 79 (2)	3–1 (2), 3–7 (1)
Finland	11. FIhi	60.34	25.24	2	1	9 (1), 37 (1), 38 (1)	3–2 (3)
	12. FIla	61.06	25.05	1	–	9 (1)	3–2 (1)
	13. FIma	61.38	26.83	3	–	9 (1), 83 (2)	3–2 (3)
	14. FIot	64.14	27.09	2	–	9 (1), 77 (1)	3–2 (1), 3–3 (1)
	15. FIpa	64.40	27.86	8	2	8 (4), 9 (2), 84 (2), 85 (2)	3–2 (4), 3–3 (6)
	16. FIpi	60.20	24.89	3	2	9 (1), 38 (2), 89 (2)	3–2 (5)
	17. FIsi	61.58	29.57	3	2	9 (2), 86 (2), 87 (1)	3–2 (5)
	18. FIsk	59.93	23.32	1	–	78 (1)	3–4 (1)
	19. FItu	62.81	28.49	2	–	9 (2)	3–2 (2)
	20. FItv	59.84	23.24	7	2	9 (6), 95 (2), 96 (1)	3–2 (9)
	21. FIuu	60.20	25.18	1	1	9 (2)	3–2 (2)
	22. FIva	60.35	25.08	1	1	39 (1), 40 (1)	3–2 (2)
	23. FIve	60.21	24.84	1	1	38 (1), 88 (1)	3–2 (2)
	24. FIvi	60.22	25.03	8	11	9 (5), 97 (5), 98 (4), 99 (2), 100 (2), 101 (1)	3–2 (16), 3–3 (3)
France	25. FR	43.95	7.52	1	–	46 (1)	3–1 (1)
	26. FRaz	47.35	0.87	10	–	1 (4), 45 (2), 75 (1), 76 (3)	3–1 (5), 3–7 (5)
	27. FRla	45.23	5.85	5	–	1 (5)	3–1 (5)
	28. FRmo	46.08	6.69	11	3	1 (7), 2 (4), 8 (3)	3–1 (11), 3–3 (3)
	29. FRmove	46.09	6.66	1	–	74 (1)	3–3 (1)
Germany	30. GEbb	49.96	8.95	2	1	1 (1), 45 (1), 94 (1)	3–1 (1), 3–7 (2)
	31. GEbz	51.18	14.43	1	4	50 (4), 93 (1)	3–7 (5)
	32. GEer	48.36	9.19	3	–	1 (2), 68 (1)	3–1 (2), 3–3 (1)
	33. GEhe	51.87	11.27	14	–	1 (6), 8 (6), 49 (2)	3–1 (6), 3–3 (8)
	34. GElb	51.10	14.67	1	4	1 (2), 90 (1), 91 (1), 92 (1)	3–1 (3), 3–2 (2)
	35. GEnt	53.97	10.77	4	–	70 (4)	3–7 (4)
	36. GEri	49.87	9.97	13	–	1 (6), 69 (1), 70 (4), 71 (2)	3–1 (7), 3–7 (6)
	37. GEse	48.10	9.64	4	–	45 (4)	3–7 (4)
	38. GEst	53.99	10.80	6	–	1 (2), 72 (2), 73 (2)	3–1 (4), 3–7 (2)
	39. GEwi	47.62	11.59	15	–	45 (7), 50 (8)	3–7 (15)
Italy	40. ITar	45.50	8.34	5	–	55 (5)	3–1 (5)
	41. ITca	45.12	7.49	15	–	52 (12), 53 (2), 54 (1)	3–1 (15)
	42. ITgo	45.76	8.41	6	–	53 (3), 58 (1), 59 (1), 60 (1)	3–1 (5), 3–3 (1)
	43. ITin	45.75	8.47	2	–	53 (1), 62 (1)	3–1 (2)
	44. ITla	45.52	8.37	2	–	56 (1), 57 (1)	3–1 (2)
	45. ITng	45.65	8.40	2	–	52 (1), 53 (1)	3–1 (2)
	46. ITqu	45.86	8.36	2	–	53 (1), 61 (1)	3–1 (2)
	47. ITso	45.79	8.91	7	–	8 (1), 53 (1), 60 (1), 63 (4)	3–1 (1), 3–3 (2), 3–4 (4)
Kyrgyzstan	48. KIR	42.71	77.70	1	–	9 (1)	3–2 (1)
Netherlands	49. NLut	52.09	5.12	10	–	30 (1), 45 (9)	3–7 (9), 3–5 (1)
Poland	50. PLka	52.27	20.46	5	–	9 (4), 51 (1)	3–2 (5)
	51. PLpu	52.59	21.46	2	–	9 (1), 13 (1)	3–2 (1), 3–4 (1)
	52. PLwa	52.23	21.01	5	–	14 (5)	3–2 (5)
Romania	53. ROcl	46.80	23.52	3	–	3 (2), 36 (1)	3–2 (1), 3–6 (2)
	54. ROcmk	46.77	23.59	1	–	3 (1)	3–6 (1)
	55. ROst	46.10	25.87	3	–	34 (3)	3–6 (3)
	56. ROtu	46.57	23.78	1	–	44 (1)	3–3 (1)
	57. ROva	45.22	28.31	1	–	35 (1)	3–2 (1)
	58. ROvo	46.64	25.63	2	–	3 (2)	3–6 (2)
Russia	59. RUac	54.86	83.10	11	1	8 (1), 13 (5), 23 (2), 24 (1), 25 (2), 30 (1)	3–2 (3), 3–3 (1) 3–4 (7), 3–5 (1)
	60. RUar	51.78	87.27	2	–	13 (1), 29 (1)	3–2 (1), 3–4 (1)
	61. RUbo	50.60	36.02	5	1	3 (2), 42 (1), 43 (3)	3–3 (4), 3–6 (2)
	62. RUbu	55.15	83.80	1	–	3 (1)	3–6 (1)
	63. RUgo	55.13	83.92	1	–	33 (1)	3–3 (1)
	64. RUlu	67.15	32.40	5	–	9 (5)	3–2 (5)
	65. RUma	54.77	83.09	2	1	9 (1), 31 (1), 32 (1)	3–2 (2), 3–6 (1)
	66. RUmo	55.76	37.62	4	–	9 (2), 14 (2)	3–2 (4)
	67. RUno	55.04	82.93	3	2	13 (2), 27 (2), 28 (1)	3–4 (5)
	68. RUpe	59.88	29.87	5	–	8 (1), 41 (4)	3–2 (4), 3–3 (1)
Sweden	69. SEbr	56.08	14.47	1	–	18 (1)	3–7 (1)
	70. SEhu	55.84	13.97	1	–	17 (1)	3–1 (1)
	71. SEjo	58.67	16.69	1	1	21 (1), 22 (1)	3–1 (2)
	72. SEka	56.21	14.83	2	–	45 (2)	3–7 (2)
	73. SEkl	57.14	15.13	1	1	19 (2)	3–7 (2)
	74. SEkr	55.70	13.48	5	–	5 (1), 6 (4)	3–1 (5)
Slovenia	75. SL	46.47	15.80	1	–	8 (1)	3–3 (1)
Spain	76. SP	42.63	–0.55	2	–	1 (2)	3–1 (2)
Switzerland	77. CHbu	46.48	6.26	2	–	1 (1), 26 (1)	3–1 (2)
	78. CHch	46.41	6.16	1	–	20 (1)	3–1 (1)
	79. CHge	46.44	6.23	1	–	20 (1)	3–1 (1)
	80. CHje	46.99	9.56	8	–	66 (6), 67 (2)	3–1 (2), 3–7 (6)
	81. CHpi	46.42	9.22	3	1	53 (1), 64 (1), 65 (2)	3–1 (1), 3–3 (3)
	82. CHsa	46.46	9.19	3	–	53 (3)	3–1 (3)
Ukraine	83. UA	51.03	28.45	1	1	9 (1), 15 (1)	3–2 (2)
	84. UAgo	46.47	32.30	2	–	9 (2)	3–2 (2)
	85. UAka	49.72	31.52	3	–	8 (1), 9 (2)	3–2 (2), 3–3 (1)
	86. UAki	50.41	30.47	2	1	8 (2), 16 (1)	3–2 (1), 3–3 (2)
	87. UAri	51.22	27.23	1	–	9 (1)	3–2 (1)
	88. UAvo1	51.62	23.82	3	–	9 (2), 10 (1)	3–2 (2), 3–3 (1)
	89. UAvo2	51.59	23.76	3	2	9 (3), 11 (1), 12 (1)	3–2 (4), 3–7 (1)
	90. UAvo3	51.48	23.89	1	–	13 (1)	3–4 (1)
	91. UAvo4	51.25	25.73	1	–	9 (1)	3–2 (1)
	92. UAvo5	51.54	23.85	1	–	9 (1)	3–2 (1)
	93. UAvo6	51.57	23.75	2	–	9 (1), 14 (1)	3–2 (2)
USA	94. USca	42.38	–11.36	2	–	9 (1), 45 (1)	3–2 (1), 3–7 (1)
	95. USma	–	–	2	–	7 (2)	3–1 (2)
In total				371	48		

**Figure 2:**
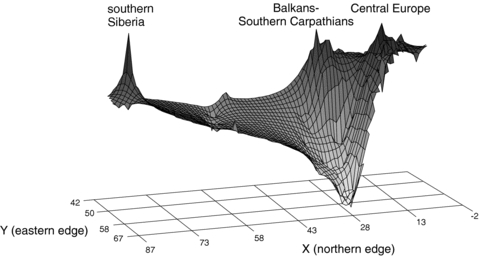
Genetic landscape; *y*-axis represents north–south dimension (degrees latitude), *x*-axis east–west dimension (degrees longitude), and *z*-axis diversity (peaks locate highest diversities).

The host populations were strongly structured (Φ_ST_ = 0.54 ± 0.34, mean ± SD) and genetically diverse, though genetic diversity varied considerably among populations (Table S3). Haplotype and nucleotide diversities of host populations were *h* = 0.52 ± 0.38 and π = 0.0015 ± 0.0017 (mean ± SD), respectively. Genetic and geographic distances correlated weakly (Mantel test, *r* = 0. 2429, *P* = 0.001), indicating weak isolation by distance. In the genetic landscape, genetic diversity was highest in Central Europe, the Balkan–Southern Carpathian region and Siberia, and lowest in the north (Fig. 2).

**Figure 3:**
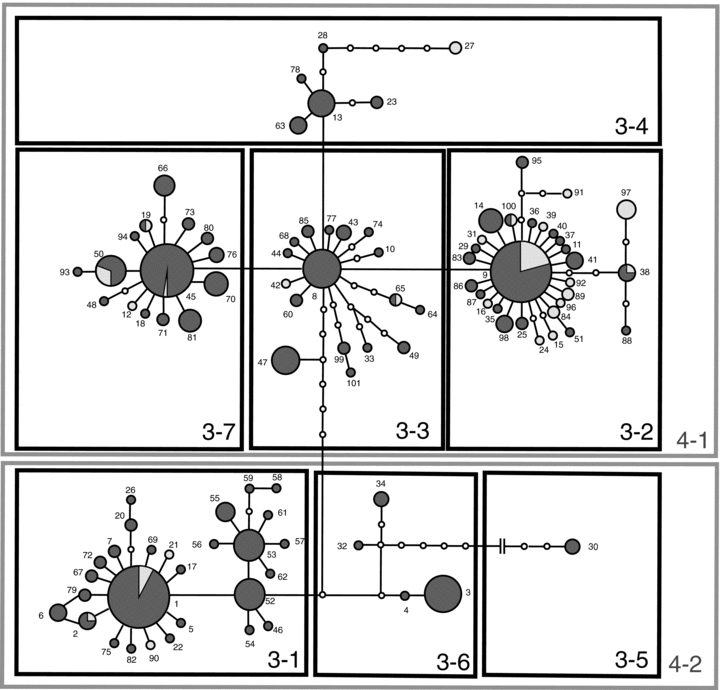
Haplotype network of combined data showing third- and fourth-level haplogroups with group numbers. Small numbers refer to haplotypes; size of haplotype is proportional to number of individuals. Each line, regardless of its length, is a single mutational step, and an open circle is a hypothetical haplotype. Branch between haplogroups 3–5 and 3–6 includes 15 mutational steps (cut for the figure). Hosts (macrogyne queen or worker) in dark gray and parasites (microgyne queen) in light gray.

The haplotypes clustered into haplogroups at four levels (Fig. 3), the fifth level consisting of the total network. The fourth level divided the network into two main lineages, both containing third-level groups that indicated a west–east division of haplogroups, with a division line running from eastern Scandinavia to the northern coast of the Adriatic Sea (Fig. 1). Haplogroups 3–1 (within 4–2) and 3–7 (4–1) were found only west of the division line (except one haplotype of group 3–7 in the eastern population UAvo2), and haplogroups 3–2 (4–1) and 3–6 (4–2) occurred only east of the line. In Central Europe, at and west of the division line, a mix of western and eastern haplogroups was evident. There, haplogroups 3–3 and 3–4 (4–1) with an eastern weight of distribution were detected west of the division line, but not in the westernmost Europe and Scandinavia. The only haplogroup found both in the westernmost continental Europe and Siberia (3–5 with a single, rare haplotype) deviated clearly from the other haplogroups, having a minimum of 15 mutational steps to its closest-neighbor haplogroup (3–6). The samples from the USA included both western and eastern haplogroups (Figs. 1 and 3, Tables S1 and S2): Maine had a local (sampled only in this area) haplotype (h7) in the western group 3–1, whereas those from Massachusetts were common, central, and widespread haplotypes of the western group 3–7 and the eastern group 3–2.

In the analysis of recent population expansion, haplogroups 3–1, 3–2, and 3–7 had significant negative Fu's *F_S_* values (Table 2). Each haplogroup fitted the stepwise demographic expansion model, though 3–6 had a poor fit to the model. The mismatch distribution was unimodal for all haplogroups except 3–3 that had a bimodal distribution (Table 2). For the bimodal mismatch distribution, the analysis used the right peak to define tau (τ). This estimate of the mutational time since population expansion varied among haplogroups from one to five mutations and their CI were wide. According to the net distances using the mutation rate of 1.15% Ma^−1^, the third-level haplogroups diverged 43–522 ka BP (Table 3). The divergence estimates with the mutation rates of 4% Ma^−1^ and 5% Ma^−1^ were 13–150 ka BP and 10–120 ka BP, respectively.

**Table 2:** Indicators of population expansion for haplogroups. Number of individuals (*n*), Fu's *F_S_* value and its significance (*P*), fit to stepwise demographic expansion model, modality of mismatch distribution (number of peaks), tau (τ) mutational time since population expansion of each haplogroup as pairwise nucleotide differences and 95% confidence intervals (CI)

			**Mismatch distribution**		
				
**Haplogroup**	***n***	**Fu's *F_S_* (*P*)**	**Fit to model (*P*)**	**Peaks**	**τ (95% CI)**
3–1	110	−20.727 (0.000)	0.965	1	1.859 (0.363–3.539)
3–1*	76	−13.399 (0.003)	0.766	1	1.717 (0.000–4.426)
3–2	80	−16.670 (0.000)	0.162	1	1.180 (0.758–1.697)
3–3	48	−2.754 (0.130)	0.162	2	5.021 (0.375–10.242)
3–4	18	−0.974 (0.209)	0.759	1	1.137 (0.000–2.443)
3–6	24	1.152 (0.763)	0.063	1	1.464 (0.359–3.500)
3–7	84	−9.694 (0.000)	0.188	1	1.344 (0.875–1.883)

* exclusion of Italian samples from the analysis.

**Table 3:** Jukes–Cantor net distances (*d*) between third-level haplogroups, their 95% confidence intervals (CI) and time parameter (*t*) in kilo years (ka) with 95% CI according to three different mutation rates (1.15%, 4%, and 5% Ma^−1^)

**Comparison**	**Net distance (CI)**	**Divergence time *t* (CI) ka Mutation rate 1.15% Ma^−1^**	**4% Ma^−1^**	**5% Ma^−1^**
3–1 vs. 3–3	0.0020 (0.0020–0.0060)	174 (89–259)	50 (26–75)	40 (20–60)
3–1 vs. 3–6	0.0039 (0.0001–0.0079)	174 (3–344)	50 (1–99)	40 (1–79)
3–2 vs. 3–3	0.0020 (0.0000–0.0030)	43 (0–129)	13 (0–37)	10 (0–30)
3–3 vs. 3–4	0.0020 (0.0000–0.0030)	43 (0–129)	13 (0–37)	10 (0–30)
3–3 vs. 3–6	0.0039 (0.0011–0.0089)	217 (47–388)	63 (14–112)	50 (11–89)
3–3 vs. 3–7	0.0020 (0.0000–0.0030)	43 (0–129)	13 (0–37)	10 (0–30)
3–5 vs. 3–6	0.0059 (0.0061–0.0179)	522 (266–777)	150 (77–224)	120 (61–179)

### Parasitized populations

The queens formed two size groups without medium-sized individuals. The mean (± SD) mesosoma length of the macrogynes (the host) was 2.12 ± 0.064 mm (min–max = 1.95–2.31 mm; *n* = 167) and that of the microgynes (the inquiline) 1.65 ± 0.055 mm (1.55–1.76 mm; *n* = 35).

All haplogroups except 3–5 and 3–6 were encountered in parasitic individuals (Fig. 3; Table 4, S1, and S2). Genetic differentiation between the host and parasite varied substantially among populations, but in 84% of the parasitized nests, the host and parasite had different haplotypes (Table 4). Local host and parasite populations differed by zero to seven (2.5 ± 1.7, mean ± SD) mutational steps. The parasite populations differed genetically from their local host populations (Φ_ST_ = 0.34 ± 0.32, mean ± SD; min–max = 0.04–0.86; Table 4) and from other parasite populations (Φ_ST_ = 0.70 ± 0.35, mean ± SD; min–max = 0–1). The parasite populations were genetically less diverse than those of the host (Table S3).

**Table 4:** Parasitized populations (*n* = 24; capital letters refer to country, lowercase letters to area within country; detailed population and haplotype information in Table 1 and S1), numbers of sequenced hosts and parasites in each population, haplotypes of hosts and parasites with haplotype identification numbers, average number of pairwise nucleotide differences between local hosts and parasites, Φ_ST_-values between host and parasite subpopulations, and nest-level comparisons (37 nests with both host and parasite) of haplotypes between parasite and host (numbers of identical and different haplotypes)

	**No. of sequenced**		**Haplotypes**				**Host/parasite haplotype**	
					
**Population**	**Hosts**	**Parasites**	**Host**	**Parasite**	**Nucleotide Differences**	**Φ_ST_**	**Identical**	**Different**
ENdose	1	1	1	1	0	–	1	0
FIhi	2	1	37, 38	9	2	–	0	1
FIpa	8	2	8, 9, 85	84	2	0.65	0	2
FIpi	3	2	9, 38	89	3	0.57	0	2
FIsi	3	2	86, 87	9	1	0.17	0	2
FItv	7	2	9, 95	9, 96	1.36	0.04	0	2
FIuu	1	1	9	9	0	–	–	–
FIVa	1	1	40	39	2	–	0	1
Five	1	1	88	38	2	–	0	1
FIvi	8	11	98, 99, 100, 101	9, 97, 100	4.59	0.34	1	6
FRmo	11	3	1, 2, 8	1, 2	2.33	−0.04	1	2
GEbb	2	1	1, 94	45	4.5	–	0	1
GEbz	1	4	93	50	1	–	0	1
Gelb	1	4	1	1, 90, 91, 92	4.75	–	1	0
RUac	11	1	8, 13, 23, 25, 30	24	5.82	–	0	1
RUbo	5	1	3, 43	42	5.2	–	0	1
RUma	2	1	9, 32	31	5	–	0	1
RUno	3	2	13, 28	27	6.33	0.86	0	2
SEjo	1	1	22	21	2	–	0	1
SEkl	1	1	19	19	0	–	1	0
CHpi	3	1	53, 64, 65	65	3.67	–	1	0
UA	1	1	9	15	2	–	0	1
UAki	2	1	8	16	2	–	0	1
UAvo2	3	2	9	9, 12	1.83	0.15	0	2
Total	82	48	45	31			6 (16%)	31 (84%)
Mean ± SD					2.5 ± 1.7	0.34 ± 0.32		

An AMOVA based on pairwise differences and frequencies of haplotypes resulted in no, or small and statistically nonsignificant differences between the queen morphs (Table 5A). An AMOVA based on haplotype frequencies only, and grouping at the highest hierarchical level by queen morph, yielded a similar result. When grouping by population, however, ca. 22% (*P* = 0.00000) of the genetic variation was explained by queen morph within populations (Table 5B).

**Table 5:** Analysis of molecular variance (AMOVA) based on (A) pairwise differences and frequencies of haplotypes, and (B) only haplotype frequencies; levels of significance from 20,022 random permutations

**(A)**	**Pairwise differences and frequencies of haplotypes**				
				
**Grouping**	**Source of variation**	**df**	**Fixation index**	**Percentage of total variation**	***P*-value**
**Queen morph**	Between queen morphs	1	Φ_CT_ = –0.00727	–0.73	0.52897
	Among populations within queen morphs	46	Φ_SC_ = 0.41241	41.54	0.00000
	Within queen morphs within populations	82	Φ_ST_ = 0.40814	59.19	0.00000
	Total	129			
**Population**	Among populations	23	Φ_CT_ = 0.37574	37.57	0.00000
	Between queen morphs within populations	24	Φ_SC_ = 0.06494	4.05	0.11093
	Within queen morphs within populations	82	Φ_ST_ = 0.41628	58.37	0.00000
	Total	129			

**(B)**	**Haplotype frequencies only**				
				
**Grouping**	**Source of variation**	**df**	**Fixation index**	**Percentage of total variation**	***P*-value**
**Queen morph**	Between queen morphs	1	Φ_CT_ = –0.00246	–0.25	0.44082
	Among populations within queen morphs	46	Φ_SC_ = 0.31901	31.98	0.00000
	Within queen morphs within populations	82	Φ_ST_ = 0.31733	68.27	0.00000
	Total	129			
**Population**	Among populations	23	Φ_CT_ = 0.10450	10.45	0.00025
	Between queen morphs within populations	24	Φ_SC_ = 0.24077	21.56	0.00000
	Within queen morphs within populations	82	Φ_ST_ = 0.32010	67.99	0.00000
	Total	129			

## Discussion

To study the mtDNA differentiation between the *Myrmica rubra* macrogyne and its microgyne parasite, we appraised two competing hypotheses, the no-speciation versus incipient-speciation hypothesis. The parasite had both morph-specific and shared haplotypes with its host, which is in concordance with the results of Steiner et al. (2006) and Vepsäläinen et al. (2009). Locally, the parasite usually belonged to the same mtDNA haplogroup as its host; consequently, the host and parasite had a common phylogeographic structure. This is compatible with the AMOVA comparison of the queen morphs, where the groups of all hosts and all parasites explained none of the total genetic variation, in agreement with the synonymization of *M. microrubra* with *M. rubra* by Steiner et al. (2006). However, the local host and the parasite usually had different haplotypes, which fits in with the incipient-speciation hypothesis. Notably, the haplotype frequencies of the local host and parasite populations differed, and in AMOVA queen morph explained 22% of the genetic variation. This local, population-level differentiation in haplotype frequencies is a general pattern, which further supports our incipient-speciation hypothesis—though we did not detect genetic differentiation in all populations, perhaps owing to small sample size. Anyhow, if sympatric speciation is taking place, it is most likely in its early phase, not yet visible as lineage sorting of the mtDNA between the morphs.

Since the local host and parasite populations were more similar to each other than either one was to another population of its own morph, and shared a phylogeographic structure, we combine them to discuss our phylogeographic results. To compare the single-refugium and multiple-refugia hypotheses, we next examine the haplotype constitution of populations. The haplotypes of the western and eastern populations belonged predominantly to different haplogroups. This implies historical vicariance between the haplogroups, and different colonization routes, which is compatible with the multiple-refugia hypothesis. A similar west–east phylogeographic structure is found in many European taxa (Taberlet et al. 1998; Hewitt 2004), in insects, for example, butterflies (Schmitt and Krauss 2004; Wahlberg and Saccheri 2007) and ants (Goropashnaya et al. 2004b; Pusch et al. 2006; Schlick–Steiner et al. 2007). Also *Betula* birches have western and eastern lineages, and their haplogroups are—like many of the *M. rubra* haplogroups—widely distributed (Maliouchenko et al. 2007). Since *M. rubra* is common in birch forests, birches and ants may have shared both glacial refugia and postglacial recolonization routes.

Previous studies have indicated that several Pleistocene ant refugia existed in the Palearctic (Goropashnaya et al. 2004a, b, 2007; Pusch et al. 2006; Schlick–Steiner et al. 2006, 2007; Beibl et al. 2007), but the locations of the refugia and routes of postglacial colonization have remained rather unclear. We found the highest areal genetic distances, typical of refugia (Hewitt 1996), in southern Siberia (H, Fig. 1), the Balkan–Southern Carpathian region (C, Fig. 1), and in Central Europe (Fig. 2). Since high areal genetic distances may, however, also result from old lineages having met during postglacial recolonization (Comps et al. 2001; Petit et al. 2003), we next evaluate the locations of *M. rubra*'s plausible refugia in more detail by looking at the distribution of their haplotypes.

Because several haplotypes and haplogroups of *M. rubra* had wide distributions, reconstruction of their past processes is complicated. Nevertheless, to find indications of potential refugia and recolonization routes, we applied the following reasoning. Haplotypes that are frequent or central in a haplogroup are usually considered older than those that are rare or lie at the fringes of a haplogroup (Donnelly and Tavare 1986; Castelloe and Templeton 1994). Thus, when found within a potential refugium, a central, common haplotype would support the interpretation that *M. rubra* survived the glaciation in that specific refugium, and spread from there to its present-day areas of occurrence. On the other hand, many local haplotypes within a potential refugium would also imply a refugium, but from where no expansion has taken place (Hewitt 2000).

In the western haplogroup 3–1, the central, common haplotype (h1) occurred throughout western and Central Europe. Within potential refugia, we collected h1 in Spain (A, Fig. 1) and southern France (D, Fig. 1; Table 6). Since it is unlikely that postglacial colonization by *M. rubra*—a relatively cold-adapted North Palearctic species (Kipyatkov 2001; Czechowski et al. 2002)—occurred southward, these results indicate Iberia (A, Fig. 1) as the most probable refugium and a source of recolonization of haplogroup 3–1. Furthermore, the local Andorran haplotype (h47) also points to an Iberian refugium (see below). Without doubt, Iberia has served as a refugium for many taxa (Hewitt 1999; Gómez and Lunt 2007), though not recognized earlier for the ants (Goropashnaya et al. 2004a, b, 2007; Pusch et al. 2006; Beibl et al. 2007; Schlick–Steiner et al. 2007).

**Table 6:** Occurrence of haplotypes within potential refugia (shown in Fig. 1); populations (numbers in Table 1), number of haplotypes and total number of individuals (*n*), haplogroup with central and local haplotypes (haplogroup/haplotype) in each refugium with numbers of individuals (*n*)

**Potential refugium**	**Populations**	**Haplotypes (*n*)**	**Central haplotypes (*n*)**	**Local haplotypes (*n*)**
A. Iberia	1, 76	2 (14)	3–1/1 (2)	3–3/47 (12)
B. The Apennines	40–47	13 (41)	3–3/8 (1)	3–1/52 (13), 54 (1), 55 (5), 56 (1), 57 (1), 58 (1), 59 (1), 61 (1), 62 (1); 3–3/60 (2); 3–4/63 (4)
C. The Balkans–Southern Carpathians	4, 53–56, 58	5 (22)	3–6/3 (16)	3–2/36 (1); 3–3/44 (1); 3–6/4 (1), 34 (3)
D. Southern France	27	1(5)	3–1/1 (5)	
G. Middle Asia	48	1 (1)	3–2/9 (1)	
H. Siberia	59–60, 62–63, 65, 67	14 (24)	3–2/9 (1); 3–3/8 (1); 3–4/13 (8); 3–6/3 (1)	3–2/24 (1), 25 (2), 29 (1), 31 (1); 3–3/33 (1); 3–4/23 (2), 27 (2), 28 (1); 3–6/32 (1)

Also within haplogroup 3–1, the 11 Italian haplotypes (h52, h53, and the haplotypes radiating from them) all differentiated from the central starlike set of the other 17 haplotypes in the same haplogroup (Fig. 3; Table 6). Only one Italian haplotype (h53) was found elsewhere, high in the Swiss Alps, next to the Italian border. Evidently, the Apennine Peninsula (B, Fig. 1) has been a refugium for *M. rubra* and favored regional differentiation of haplogroup 3–1, though without successful colonization beyond the Alps. The Alps have, indeed, been an effective dispersal barrier of many taxa (Hewitt 2000), for example, the meadow grasshopper *Chorthippus parallelus* (Hewitt 1996) and the ant *Tetramorium moravicum* (Schlick–Steiner et al. 2007).

The other western haplogroup 3–7 is more puzzling, as it was absent from all putative refugia. This may result from insufficient sampling, extinction of the haplogroup within a refugium, or survival in a more northern refugium. Since *M. rubra* is cold-tolerant and its distribution extends north, close to the forest-tundra zone (Kipyatkov 2001; Czechowski et al. 2002), it may have survived the last glaciation also north of the southern European peninsulas, as have some mammal species (Sommer and Nadachowski 2006). As the central haplotype (h45) of the group was present in southern France (D, Fig. 1) and Austria, a refugium may have situated there or in adjacent areas, where some forest stands were present during the LGM (Willis and van Andel 2004). We did not, however, consider Austria as potential refugium since the forest stands there were mainly coniferous and, thus, marginal habitat for *M. rubra* (Czechowski et al. 2002).

The geographically central haplogroup 3–3 was found from Siberia to Andorra. Its central, common haplotype (h8) occurred in two potential refugia: southern Siberia (H, Fig. 1) and the Apennine Peninsula (B, Fig. 1; Table 6). Because the distribution of h8 is so wide, it is difficult to deduce whether postglacial recolonization north originated from the Apennines or more east. The eastern haplotypes (see below) in eastern Fennoscandia, however, suggest that recolonization of group 3–3 likely occurred from an eastern refugium rather than the Apennines. Also, h8 may be new to the Apennines, particularly if some of the postglacial recolonization of *M. rubra* took place from the Balkan–Carpathian region (C, Fig. 1) to Italy as in some tree species (Hewitt 1999)—our sample (h8) from Slovenia, at the northwestern fringe of the Balkans, would support such an interpretation. Finally, the location of the geographically distant and differentiated population in Andorra, at an elevation of over 1500 m a.s.l., also indicates the Iberian Peninsula (A, Fig. 1) as a refugium of haplogroup 3–3, but no recolonization of more northern areas from there.

The eastern haplogroup 3–2 and its central, common haplotype (h9) occurred from southern Siberia (H, Fig. 1) and Middle Asia (G, Fig. 1) to eastern Europe, north of the Carpathians, and eastern Fennoscandia (Table 6). The most probable refugium and source area of recolonization of haplogroup 3–2 has been in the Caspian–Caucasus region (F, Fig. 1) or possibly more east, in Middle Asia or southern Siberia—including, as suggested for *Formica* ants (Goropashnaya et al. 2004b)—the south Siberian mountains, such as the Altai Mountains (H, Fig. 1).

Of the rarer haplogroups, 3–6 had a clearly southeastern–eastern distribution. The central, common haplotype (h3) and several local haplotypes (including some of the other haplogroups) occurred in the Balkan–Southern Carpathian region (C, Fig. 1; Table 6), which indicates that this area served as refugium for *M. rubra*. The area has been climatically stable through the Quaternary (Tzedakis et al. 2002), and it has been considered as one of the main refugia for European biota during the last glaciation (Taberlet et al. 1998; Hewitt 1999). In the ants, the Balkan–Carpathian region has probably served as a refugium for *T. moravicum* (Schlick–Steiner et al. 2007), the *Formica rufa* group ants (Goropashnaya et al. 2004a), and the western group of *F. pratensis* (Goropashnaya et al. 2004b). We cannot, however, exclude the Caspian–Caucasus region (F, Fig. 1), Middle Asia (G, Fig. 1), or southern Siberia (H, Fig. 1) as refugia in their own right for haplogroup 3–6 (Table 6).

Haplogroup 3–4 is the most eastern in our samples, with most of its central, common haplotype (h13)—and notable differentiation, typical of refugia—found in Siberia (H, Fig. 1; Table 6). Probably the haplogroup has either existed in several refugia or gone through extensive postglacial recolonization from a single refugium. The rare haplogroup 3–5, very differentiated from the other haplogroups, had only one haplotype, found in southern Siberia (H, Fig. 1) and western Europe. Since the haplogroup may exist in nonsampled areas, it is difficult to infer anything about its distribution and history. However, it may also be an old, formerly extensively distributed haplogroup, or perhaps a human-induced introduction between remote sites.

Our results on the distribution of the haplotypes thus support the hypothesis that *M. rubra* survived the ice age in several refugia, scattered over an extensive area from Iberia in the west to Siberia in the east. As also suggested by the multiple-refugia hypothesis, postglacial recolonization of the species to the present areas of distribution occurred along several routes. These postglacial colonization routes, headed northward from Iberia and the Balkan–Carpathian region, formed a wide secondary contact zone of high genetic diversity in Central Europe (seen also in Fig. 2). Such contact zones are commonly found in many taxa (Hewitt 2004), for example, in *Temnothorax* ants (Pusch et al. 2006). The southwestern border of the eastern haplogroups 3–2 and 3–6 was in the Balkan Peninsula, which is a contact zone for western and eastern lineages of the ant *T. moravicum* (Schlick–Steiner et al. 2007) and different genetic lineages of *Messor* cf. *structor* (Schlick–Steiner et al. 2006).

*Myrmica rubra* has been introduced from Europe to North America (Groden et al. 2005; Wetterer and Radchenko 2010), where it was first reported in Massachusetts in the early 20th century (Wheeler 1908). Since then it has spread to southeastern Canada and northwestern USA (Groden et al. 2005; Wetterer and Radchenko 2010). In this study, the two samples from Maine had the same local haplotype (h7), which belonged to the western haplogroup 3–1. The two samples from Massachusetts, in contrast, had widespread haplotypes, h45 of the western haplogroup 3–7 and h9 of the eastern haplogroup 3–2. Since all three haplogroups were present in the contact zone in Germany, an introduction may have originated from there.

After each Pleistocene glaciation, populations often experienced demographic and spatial expansions that left genetic imprints on their population structure (Rogers and Harpending 1992; Hewitt 1996). Haplogroups 3–1, 3–2, and 3–7 had a starlike structure, which is indicative of a recent population expansion (Rogers and Harpending 1992) and has been found in many taxa, including several European ant species (Goropashnaya et al. 2004a,b; Pusch et al. 2006). Recent population expansion of haplogroups 3–1, 3–2, and 3–7 was also implied by our results of the mismatch distributions and the significant negative Fu's *F_S_* values. Such negative values may, however, also result from genetic hitchhiking and selection (Fu 1997).

If the assumption that the haplogroups expanded after the LGM is correct and the mutation-rate estimate of 4–5% Ma^−1^ based on this assumption holds, then most third-level haplogroups diverged during and possibly after the last glaciation, in the Holocene–Late Pleistocene (Svendsen et al. 2004). Even with a more conservative mutation rate of 1.15% Ma^−1^ (Brower 1994), the third-level haplogroups in 4–1 would still have diverged in the Holocene–Late Pleistocene. Correspondingly, the conservative mutation rate would imply earlier divergence between the third-level haplogroups in 4–2, and between 4–1 and 4–2, in the Late–Middle Pleistocene. Therefore, it seems plausible that the diversification of the third-level haplogroups of *M. rubra* has occurred during the last glaciation and the subsequent postglacial expansion.

## Supporting information

**Table S1.** Genbank accession numbers for COI and Cyt b gene fragments, sample (identification) number, sampling location, haplotype number, and sample collector; sample number includes nest number, R = host, m = parasite, q = queen, w = worker, population code (capital letters refer to country, lowercase letters to areawithin country), and asterisk (*) indicates that host and parasite are from the same nest despite of different nest numbers.

**Table S2.** List of haplotypes and their haplogroups of combined COI and Cyt b gene fragments and populations (capital letters refer to country, lowercase letters to area within country) where haplotype was detected, with numbers of individuals in parentheses.

**Table S3.** Genetic diversity estimates for host and parasite populations. Population (capital letters refer to country, lowercase letters to area within country), numbers of hosts and parasites in each population, haplotype diversity (*h* ± standard deviation), genetic diversity (π ± standard deviation) for host and parasite populations with more than one individual. Asterisk (*) indicatesmean values for parasitised host populations.
