# Supplementary material for: Phylogeography of the ant Myrmica rubra and its inquiline social parasite
Source: Ecol Evol. 2011 Sep;1(1):46–62. doi: 10.1002/ece3.6 (PMC3287377; doi:10.1002/ece3.6)
Supplement: Supplementary file 1 [file ece30001-0046-SD1.doc]

**Supporting information**

**Table S1.** Genbank accession numbers for COI and Cyt b gene fragments, sample (identification) number, sampling location, haplotype number and sample collector; sample number includes nest number, R = host, m = parasite, q = queen, w = worker, population code (capital letters refer to country, lowercase letters to area within country), and asterisk (*) indicates that host and the parasite are from the same nest despite of different nest numbers.

**Genbank accession number**

**COI Cyt b Sample number Location Haplotype Collector**

JF778997 JF779369 325RwAN Andorra, Madriu–Perafita–Claror valley 47 A. Bernadou

JF779003 JF779375 326RwAN Andorra, Madriu–Perafita–Claror valley 47 A. Bernadou

JF779013 JF779385 327RwAN Andorra, Madriu–Perafita–Claror valley 47 A. Bernadou

JF779023 JF779395 328RwAN Andorra, Madriu–Perafita–Claror valley 47 A. Bernadou

JF779032 JF779404 329RwAN Andorra, Madriu–Perafita–Claror valley 47 A. Bernadou

JF779037 JF779409 330RwAN Andorra, Madriu–Perafita–Claror valley 47 A. Bernadou

JF779043 JF779415 331RwAN Andorra, Madriu–Perafita–Claror valley 47 A. Bernadou

JF779044 JF779416 332RwAN Andorra, Madriu–Perafita–Claror valley 47 A. Bernadou

JF779045 JF779417 333RwAN Andorra, Madriu–Perafita–Claror valley 47 A. Bernadou

JF779050 JF779422 334RwAN Andorra, Madriu–Perafita–Claror valley 47 A. Bernadou

JF779057 JF779429 335RwAN Andorra, Madriu–Perafita–Claror valley 47 A. Bernadou

JF779063 JF779435 336RwAN Andorra, Madriu–Perafita–Claror valley 47 A. Bernadou

JF779064 JF779436 3377RqAUac Austria, Achenkirch 45 R. Savolainen and K.Vepsäläinen

JF779065 JF779437 3379RwAUac Austria, Achenkirch 45 R. Savolainen and K.Vepsäläinen

JF779067 JF779439 3384RwAUac Austria, Achenkirch 45 R. Savolainen and K.Vepsäläinen

JF779068 JF779440 3386RwAUac Austria, Achenkirch 1 R. Savolainen and K.Vepsäläinen

JF779069 JF779441 3388RwAUac Austria, Achenkirch 50 R. Savolainen and K.Vepsäläinen

JF778998 JF779370 3265RqBEbr Belgium, Bryssel 48 R. Savolainen and K.Vepsäläinen

JF778999 JF779371 3266RqBEbr Belgium, Bryssel 45 R. Savolainen and K.Vepsäläinen

JF779000 JF779372 3267RqBEbr Belgium, Bryssel 45 R. Savolainen and K.Vepsäläinen

JF779001 JF779373 3268RqBEbr Belgium, Bryssel 45 R. Savolainen and K.Vepsäläinen

JF779002 JF779374 3269RqBEbr Belgium, Bryssel 45 R. Savolainen and K.Vepsäläinen

JF779004 JF779376 3270RqBEbr Belgium, Bryssel 1 R. Savolainen and K.Vepsäläinen

JF779005 JF779377 3271RqBEbr Belgium, Bryssel 45 R. Savolainen and K.Vepsäläinen

JF779006 JF779378 3272RwBEbr Belgium, Bryssel 45 R. Savolainen and K.Vepsäläinen

JF779007 JF779379 3273RqBEbr Belgium, Bryssel 1 R. Savolainen and K.Vepsäläinen

JF779008 JF779380 3274RqBEbr Belgium, Bryssel 45 R. Savolainen and K.Vepsäläinen

JF779009 JF779381 3276RqBEbr Belgium, Bryssel 45 R. Savolainen and K.Vepsäläinen

JF779010 JF779382 3277RqBEbr Belgium, Bryssel 45 R. Savolainen and K.Vepsäläinen

JF779011 JF779383 3278RwBEbr Belgium, Bryssel 45 R. Savolainen and K.Vepsäläinen

JF779012 JF779384 3279RqBEbr Belgium, Bryssel 30 R. Savolainen and K.Vepsäläinen

JF779014 JF779386 3280RqBEbr Belgium, Bryssel 1 R. Savolainen and K.Vepsäläinen

JF778877 JF779249 236RqBLvi Bulgaria, Vitosha 3 J. Leppänen

JF778878 JF779250 237RqBLvi Bulgaria, Vitosha 3 J. Leppänen

JF778879 JF779251 238RqBLvi Bulgaria, Vitosha 3 J. Leppänen

JF778880 JF779252 239RqBLvi Bulgaria, Vitosha 3 J. Leppänen

JF778881 JF779253 241RwBLvi Bulgaria, Vitosha 3 J. Leppänen

JF778882 JF779254 243RqBLvi Bulgaria, Vitosha 3 J. Leppänen

JF778883 JF779255 248RwBLvi Bulgaria, Vitosha 3 J. Leppänen

JF778884 JF779256 249RwBLvi Bulgaria, Vitosha 3 J. Leppänen

JF778885 JF779257 250RqBLvi Bulgaria, Vitosha 3 J. Leppänen

JF778886 JF779258 251RqBLvi Bulgaria, Vitosha 3 J. Leppänen

JF778887 JF779259 252RqBLvi Bulgaria, Vitosha 4 J. Leppänen

JF778888 JF779260 253RwBLvi Bulgaria, Vitosha 3 J. Leppänen

JF778919 JF779291 2977RwDKmo Denmark, Mols 1 R. Savolainen and K.Vepsäläinen

JF779199 JF779571 438RwENdore England, Dorset 81 J. Sorvari

JF779200 JF779572 439RqENdore England, Dorset 81 J. Sorvari

JF779201 JF779573 440RwENdore England, Dorset 81 J. Sorvari

JF779202 JF779574 441RwENdore England, Dorset 81 J. Sorvari

JF779203 JF779575 442RqENdore England, Dorset 81 J. Sorvari

JF779204 JF779576 443RwENdore England, Dorset 81 J. Sorvari

JF779205 JF779577 444RwENdore England, Dorset 81 J. Sorvari

JF779206 JF779578 445RqENdoki England, Dorset 1 J. Sorvari

JF779207 JF779579 446RqENdoki England, Dorset 1 J. Sorvari

JF779208 JF779580 447RwENdoki England, Dorset 1 J. Sorvari

JF779209 JF779581 448RwENdoki England, Dorset 82 J. Sorvari

JF779210 JF779582 449RqENdoki England, Dorset 1 J. Sorvari

JF779211 JF779583 450RqENdoki England, Dorset 1 J. Sorvari

JF779212 JF779584 451RwENdoki England, Dorset 1 J. Sorvari

JF779213 JF779585 452RqENdoki England, Dorset 1 J. Sorvari

JF779214 JF779586 453RwENdoki England, Dorset 1 J. Sorvari

AY280598 AY280582 RS244/98mENdose* England, Dorset, Seacombe 1 Savolainen and Vepsäläinen 2003

AY280600 AY280584 RS245/98RqENdose* England, Dorset, Seacombe 1 Savolainen and Vepsäläinen 2003

JF779194 JF779566 433RqENnoun England, Norwich 45 J. Sorvari

JF779195 JF779567 434RqENnoun England, Norwich 79 J. Sorvari

JF779196 JF779568 435RwENnoun England, Norwich 79 J. Sorvari

JF779197 JF779569 436RwENnoea England, Norwich 80 J. Sorvari

JF779198 JF779570 437RwENnoea England, Norwich 80 J. Sorvari

JF779238 JF779610 82mFIve Finland, Helsinki 38 J. Leppänen

JF779239 JF779611 82RwFIve Finland, Helsinki 88 J. Leppänen

JF779240 JF779612 86RqFIpi Finland, Helsinki 9 J. Leppänen

JF779241 JF779613 87mFIpi Finland, Helsinki 89 J. Leppänen

JF779242 JF779614 87RqFIpi Finland, Helsinki 38 J. Leppänen

JF779243 JF779615 88mFIpi Finland, Helsinki 89 J. Leppänen

JF779244 JF779616 88RwFIpi Finland, Helsinki 38 J. Leppänen

JF779245 JF779617 95mFIuu Finland, Helsinki 9 J. Leppänen

JF779246 JF779618 96RqFIuu Finland, Helsinki 9 J. Leppänen

GQ872390 JF779626 V10_1mFIvi Finland, Helsinki 97 Vepsäläinen *et al.* 2009

GQ872392 JF779627 V11_1mFIvi Finland, Helsinki 9 Vepsäläinen *et al.* 2009

GQ872393 JF779628 V11_3RwFIvi Finland, Helsinki 98 Vepsäläinen *et al.* 2009

GQ872394 JF779629 V12_1mFIvi Finland, Helsinki 9 Vepsäläinen *et al.* 2009

GQ872395 JF779630 V12_2RwFIvi Finland, Helsinki 98 Vepsäläinen *et al.* 2009

GQ872396 JF779631 V13_1mFIvi Finland, Helsinki 9 Vepsäläinen *et al.* 2009

GQ872397 JF779632 V13_2RwFIvi Finland, Helsinki 98 Vepsäläinen *et al.* 2009

GQ872398 JF779633 V14_1mFIvi Finland, Helsinki 9 Vepsäläinen *et al.* 2009

GQ872399 JF779634 V14_2RwFIvi Finland, Helsinki 98 Vepsäläinen *et al.* 2009

GQ872400 JF779635 V15_1mFIvi Finland, Helsinki 9 Vepsäläinen *et al.* 2009

GQ872374 JF779636 V2_2mFIvi Finland, Helsinki 97 Vepsäläinen *et al.* 2009

GQ872375 JF779637 V2_2RwFIvi Finland, Helsinki 99 Vepsäläinen *et al.* 2009

GQ872376 JF779638 V3_2mFivi Finland, Helsinki 97 Vepsäläinen *et al.* 2009

GQ872377 JF779639 V3_2RwFIvi Finland, Helsinki 99 Vepsäläinen *et al.* 2009

GQ872378 JF779640 V4_2mFIvi Finland, Helsinki 100 Vepsäläinen *et al.* 2009

GQ872379 JF779641 V4_2RwFIvi Finland, Helsinki 100 Vepsäläinen *et al.* 2009

GQ872381 JF779642 V5_3RwFIvi Finland, Helsinki 101 Vepsäläinen *et al.* 2009

GQ872382 JF779643 V6_2mFIvi Finland, Helsinki 97 Vepsäläinen *et al.* 2009

GQ872386 JF779644 V8_1mFIvi Finland, Helsinki 97 Vepsäläinen *et al.* 2009

JF778918 JF779290 2925RwFIla Finland, Lammi 9 R. Savolainen and K.Vepsäläinen

JF779220 JF779592 53RqFIma Finland, Mäntyharju 9 J. Leppänen

JF779221 JF779593 54RqFIma Finland, Mäntyharju 83 J. Leppänen

JF779222 JF779594 55RqFIma Finland, Mäntyharju 83 J. Leppänen

JF779191 JF779563 385RwFIot Finland, Otanmäki 9 J. Leppänen

JF779192 JF779564 387RwFIot Finland, Otanmäki 77 J. Leppänen

JF779187 JF779559 381RqFIpa Finland, Paltamo 9 J. Leppänen

JF779189 JF779561 382RwFIpa Finland, Paltamo 9 J. Leppänen

JF779223 JF779595 58RqFIpa Finland, Paltamo 8 J. Leppänen

JF779224 JF779596 59RqFIpa Finland, Paltamo 8 J. Leppänen

JF779225 JF779597 60RqFIpa Finland, Paltamo 8 J. Leppänen

JF779226 JF779598 61RqFIpa Finland, Paltamo 8 J. Leppänen

JF779227 JF779599 62mFIpa Finland, Paltamo 84 J. Leppänen

JF779228 JF779600 62RqFIpa Finland, Paltamo 85 J. Leppänen

JF779229 JF779601 63mFIpa Finland, Paltamo 84 J. Leppänen

JF779230 JF779602 63RqFIpa Finland, Paltamo 85 J. Leppänen

JF779233 JF779605 71mFIsi Finland, Parikkala 9 J. Leppänen

JF779234 JF779606 71RqFIsi Finland, Parikkala 86 J. Leppänen

JF779235 JF779607 73mFIsi Finland, Parikkala 9 J. Leppänen

JF779236 JF779608 73RwFIsi Finland, Parikkala 86 J. Leppänen

JF779237 JF779609 74RqFIsi Finland, Parikkala 87 J. Leppänen

JF778965 JF779337 3116RwFIhi Finland, Sipoo 37 R. Savolainen and K.Vepsäläinen

JF778966 JF779338 3117mFIhi Finland, Sipoo 9 R. Savolainen and K.Vepsäläinen

JF778967 JF779339 3117RwFIhi Finland, Sipoo 38 R. Savolainen and K.Vepsäläinen

JF779193 JF779565 390RwFIsk Finland, Skogby 78 J. Leppänen

JF779231 JF779603 65RwFItu Finland, Tuusniemi 9 J. Leppänen

JF779232 JF779604 66RqFItu Finland, Tuusniemi 9 J. Leppänen

AY280597 AY280581 RS201M/98mFItv* Finland, Tvärminne 9 Savolainen and Vepsäläinen 2003

AY280599 AY280583 RS201R/98RqFItv* Finland, Tvärminne 95 Savolainen and Vepsäläinen 2003

GQ872367 JF779619 T10_4RwFItv Finland, Tvärminne 9 Vepsäläinen *et al.* 2009

GQ872369 JF779620 T11_2RwFItv Finland, Tvärminne 9 Vepsäläinen *et al.* 2009

GQ872371 JF779621 T12_3RwFItv Finland, Tvärminne 9 Vepsäläinen *et al.* 2009

GQ872372 JF779622 T13_1mFItv Finland, Tvärminne 96 Vepsäläinen *et al.* 2009

GQ872373 JF779623 T13_4RwFItv Finland, Tvärminne 95 Vepsäläinen *et al.* 2009

GQ872351 JF779624 T2_3RwFItv Finland, Tvärminne 9 Vepsäläinen *et al.* 2009

GQ872353 JF779625 T3_3RwFItv Finland, Tvärminne 9 Vepsäläinen *et al.* 2009

JF778968 JF779340 3118mFIva Finland, Vantaa 39 R. Savolainen and K.Vepsäläinen

JF778969 JF779341 3118RwFIva Finland, Vantaa 40 R. Savolainen and K.Vepsäläinen

JF779172 JF779544 363RwFRaz France, Azay sur Cher 1 A. Lenoir

JF779173 JF779545 364RwFRaz France, Azay sur Cher 1 A. Lenoir

JF779174 JF779546 365RwFRaz France, Azay sur Cher 1 A. Lenoir

JF779175 JF779547 366RwFRaz France, Azay sur Cher 45 A. Lenoir

JF779176 JF779548 367RwFRaz France, Azay sur Cher 45 A. Lenoir

JF779177 JF779549 368RwFRaz France, Azay sur Cher 75 A. Lenoir

JF779178 JF779550 369RwFRaz France, Azay sur Cher 76 A. Lenoir

JF779179 JF779551 370RwFRaz France, Azay sur Cher 76 A. Lenoir

JF779180 JF779552 371RwFRaz France, Azay sur Cher 76 A. Lenoir

JF779181 JF779553 372RwFRaz France, Azay sur Cher 1 A. Lenoir

JF779182 JF779554 373RwFRla France, Lancey 1 A. Lenoir

JF779183 JF779555 374RwFRla France, Lancey 1 A. Lenoir

JF779184 JF779556 376RwFRla France, Lancey 1 A. Lenoir

JF779185 JF779557 379RwFRla France, Lancey 1 A. Lenoir

JF779186 JF779558 380RwFRla France, Lancey 1 A. Lenoir

JF778875 JF779247 151_01mFRmo* France, Morillon 1 A. Lenoir

JF778876 JF779248 153_01RqFRmo* France, Morillon 2 A. Lenoir

JF779131 JF779503 351RqFRmo France, Morillon 1 A. Lenoir

JF779134 JF779506 352RwFRmo France, Morillon 1 A. Lenoir

JF779135 JF779507 353RwFRmo France, Morillon 1 A. Lenoir

JF779141 JF779513 355RwFRmo France, Morillon 1 A. Lenoir

JF779145 JF779517 356RwFRmo France, Morillon 1 A. Lenoir

JF779158 JF779530 358RwFRmo France, Morillon 8 A. Lenoir

JF779165 JF779537 359RqFRmo France, Morillon 8 A. Lenoir

JF779170 JF779542 360RqFRmo France, Morillon 8 A. Lenoir

DQ074371 DQ074417 i882mFRmo France, Morillon 2 Steiner *et al.* 2006

DQ074372 DQ074418 i882rRqFRmo France, Morillon 2 Steiner *et al.* 2006

DQ074366 DQ074412 i883mFRmo France, Morillon 1 Steiner *et al.* 2006

DQ075937 DQ074419 i883rRqFRmo France, Morillon 2 Steiner *et al.* 2006

JF778995 JF779367 321RwFR France, Seealpen 46 R. Schultz

JF779171 JF779543 361RqFRmove France, Verchaix 74 A. Lenoir

DQ074380 DQ074426 i963rRqGEbb Germany, Babenhausen 94 Steiner *et al.* 2006

DQ074379 DQ074425 i964maGEbb Germany, Babenhausen 45 Steiner *et al.* 2006

DQ074370 DQ074416 i964rRqGEbb Germany, Babenhausen 1 Steiner *et al.* 2006

DQ074386 DQ074432 i833mbmGEbz Germany, Bautzen 50 Steiner *et al.* 2006

DQ074382 DQ074428 i833mGEbz Germany, Bautzen 50 Steiner *et al.* 2006

DQ074384 DQ074430 i839mGEbz Germany, Bautzen 50 Steiner *et al.* 2006

DQ074385 DQ074431 i840mGEbz Germany, Bautzen 50 Steiner *et al.* 2006

DQ074383 DQ074429 i840rRqGEbz Germany, Bautzen 93 Steiner *et al.* 2006

JF779140 JF779512 3555RwGEer Germany, Erpfingen 68 R. Savolainen and K.Vepsäläinen

JF779142 JF779514 3566RqGEer Germany, Erpfingen 1 R. Savolainen and K.Vepsäläinen

JF779143 JF779515 3567RwGEer Germany, Erpfingen 1 R. Savolainen and K.Vepsäläinen

JF779026 JF779398 3292RqGEhe Germany, Hedersleben 1 R. Savolainen and K.Vepsäläinen

JF779027 JF779399 3293RqGEhe Germany, Hedersleben 1 R. Savolainen and K.Vepsäläinen

JF779028 JF779400 3294RwGEhe Germany, Hedersleben 1 R. Savolainen and K.Vepsäläinen

JF779029 JF779401 3295RwGEhe Germany, Hedersleben 1 R. Savolainen and K.Vepsäläinen

JF779030 JF779402 3296RqGEhe Germany, Hedersleben 1 R. Savolainen and K.Vepsäläinen

JF779031 JF779403 3297RwGEhe Germany, Hedersleben 1 R. Savolainen and K.Vepsäläinen

JF779034 JF779406 3300RqGEhe Germany, Hedersleben 49 R. Savolainen and K.Vepsäläinen

JF779035 JF779407 3302RwGEhe Germany, Hedersleben 49 R. Savolainen and K.Vepsäläinen

JF779036 JF779408 3308RwGEhe Germany, Hedersleben 8 R. Savolainen and K.Vepsäläinen

JF779038 JF779410 3311RwGEhe Germany, Hedersleben 8 R. Savolainen and K.Vepsäläinen

JF779039 JF779411 3312RwGEhe Germany, Hedersleben 8 R. Savolainen and K.Vepsäläinen

JF779040 JF779412 3313RqGEhe Germany, Hedersleben 8 R. Savolainen and K.Vepsäläinen

JF779041 JF779413 3316RwGEhe Germany, Hedersleben 8 R. Savolainen and K.Vepsäläinen

JF779042 JF779414 3317RqGEhe Germany, Hedersleben 8 R. Savolainen and K.Vepsäläinen

JF779159 JF779531 3590RwGEnt Germany, Hemmelsdorf 70 R. Savolainen and K.Vepsäläinen

JF779160 JF779532 3594RqGEnt Germany, Hemmelsdorf 70 R. Savolainen and K.Vepsäläinen

JF779161 JF779533 3595RwGEnt Germany, Hemmelsdorf 70 R. Savolainen and K.Vepsäläinen

JF779162 JF779534 3596RwGEnt Germany, Hemmelsdorf 70 R. Savolainen and K.Vepsäläinen

DQ074367 DQ074413 i834mGElb Germany, Löbau 90 Steiner *et al.* 2006

DQ074368 DQ074414 i835mGElb Germany, Löbau 1 Steiner *et al.* 2006

DQ074369 DQ074415 i835rRqGElb Germany, Löbau 1 Steiner *et al.* 2006

DQ074387 DQ074433 i837mGElb Germany, Löbau 91 Steiner *et al.* 2006

DQ074374 DQ074420 i838mGElb Germany, Löbau 92 Steiner *et al.* 2006

JF779144 JF779516 3569RqGEri Germany, Rimpar 69 R. Savolainen and K.Vepsäläinen

JF779146 JF779518 3570RqGEri Germany, Rimpar 1 R. Savolainen and K.Vepsäläinen

JF779147 JF779519 3571RwGEri Germany, Rimpar 1 R. Savolainen and K.Vepsäläinen

JF779148 JF779520 3572RqGEri Germany, Rimpar 1 R. Savolainen and K.Vepsäläinen

JF779149 JF779521 3573RqGEri Germany, Rimpar 1 R. Savolainen and K.Vepsäläinen

JF779150 JF779522 3575RqGEri Germany, Rimpar 1 R. Savolainen and K.Vepsäläinen

JF779151 JF779523 3576RqGEri Germany, Rimpar 1 R. Savolainen and K.Vepsäläinen

JF779152 JF779524 3577RqGEri Germany, Rimpar 70 R. Savolainen and K.Vepsäläinen

JF779153 JF779525 3583RwGEri Germany, Rimpar 70 R. Savolainen and K.Vepsäläinen

JF779154 JF779526 3585RwGEri Germany, Rimpar 70 R. Savolainen and K.Vepsäläinen

JF779155 JF779527 3586RwGEri Germany, Rimpar 71 R. Savolainen and K.Vepsäläinen

JF779156 JF779528 3587RqGEri Germany, Rimpar 71 R. Savolainen and K.Vepsäläinen

JF779157 JF779529 3588RwGEri Germany, Rimpar 70 R. Savolainen and K.Vepsäläinen

JF779136 JF779508 3545RwGEse Germany, Seekirch 45 R. Savolainen and K.Vepsäläinen

JF779137 JF779509 3547RqGEse Germany, Seekirch 45 R. Savolainen and K.Vepsäläinen

JF779138 JF779510 3549RqGEse Germany, Seekirch 45 R. Savolainen and K.Vepsäläinen

JF779139 JF779511 3553RwGEse Germany, Seekirch 45 R. Savolainen and K.Vepsäläinen

JF779163 JF779535 3597RwGEst Germany, Travemünde 1 R. Savolainen and K.Vepsäläinen

JF779164 JF779536 3599RwGEst Germany, Travemünde 1 R. Savolainen and K.Vepsäläinen

JF779166 JF779538 3600RwGEst Germany, Travemünde 72 R. Savolainen and K.Vepsäläinen

JF779167 JF779539 3601RwGEst Germany, Travemünde 72 R. Savolainen and K.Vepsäläinen

JF779168 JF779540 3603RqGEst Germany, Travemünde 73 R. Savolainen and K.Vepsäläinen

JF779169 JF779541 3604RwGEst Germany, Travemünde 73 R. Savolainen and K.Vepsäläinen

JF779046 JF779418 3345RwGEwi Germany, Winkel 50 R. Savolainen and K.Vepsäläinen

JF779047 JF779419 3346RqGEwi Germany, Winkel 45 R. Savolainen and K.Vepsäläinen

JF779048 JF779420 3348RqGEwi Germany, Winkel 45 R. Savolainen and K.Vepsäläinen

JF779049 JF779421 3349RwGEwi Germany, Winkel 50 R. Savolainen and K.Vepsäläinen

JF779051 JF779423 3352RqGEwi Germany, Winkel 45 R. Savolainen and K.Vepsäläinen

JF779052 JF779424 3354RwGEwi Germany, Winkel 45 R. Savolainen and K.Vepsäläinen

JF779053 JF779425 3355RqGEwi Germany, Winkel 45 R. Savolainen and K.Vepsäläinen

JF779054 JF779426 3356RqGEwi Germany, Winkel 50 R. Savolainen and K.Vepsäläinen

JF779055 JF779427 3357RqGEwi Germany, Winkel 50 R. Savolainen and K.Vepsäläinen

JF779056 JF779428 3359RwGEwi Germany, Winkel 50 R. Savolainen and K.Vepsäläinen

JF779058 JF779430 3360RqGEwi Germany, Winkel 50 R. Savolainen and K.Vepsäläinen

JF779059 JF779431 3361RwGEwi Germany, Winkel 50 R. Savolainen and K.Vepsäläinen

JF779060 JF779432 3363RqGEwi Germany, Winkel 45 R. Savolainen and K.Vepsäläinen

JF779061 JF779433 3365RwGEwi Germany, Winkel 50 R. Savolainen and K.Vepsäläinen

JF779062 JF779434 3367RwGEwi Germany, Winkel 45 R. Savolainen and K.Vepsäläinen

JF779092 JF779464 3453RqITar Italy, Arborio 55 R. Savolainen and K.Vepsäläinen

JF779093 JF779465 3454RwITar Italy, Arborio 55 R. Savolainen and K.Vepsäläinen

JF779094 JF779466 3456RwITar Italy, Arborio 55 R. Savolainen and K.Vepsäläinen

JF779095 JF779467 3457RwITar Italy, Arborio 55 R. Savolainen and K.Vepsäläinen

JF779096 JF779468 3459RqITar Italy, Arborio 55 R. Savolainen and K.Vepsäläinen

JF779076 JF779448 3432RwITca Italy, Caselette 52 R. Savolainen and K.Vepsäläinen

JF779077 JF779449 3433RqITca Italy, Caselette 52 R. Savolainen and K.Vepsäläinen

JF779078 JF779450 3435RqITca Italy, Caselette 52 R. Savolainen and K.Vepsäläinen

JF779079 JF779451 3439RqITca Italy, Caselette 53 R. Savolainen and K.Vepsäläinen

JF779081 JF779453 3440RqITca Italy, Caselette 52 R. Savolainen and K.Vepsäläinen

JF779082 JF779454 3441RqITca Italy, Caselette 52 R. Savolainen and K.Vepsäläinen

JF779083 JF779455 3442RqITca Italy, Caselette 52 R. Savolainen and K.Vepsäläinen

JF779084 JF779456 3443RqITca Italy, Caselette 52 R. Savolainen and K.Vepsäläinen

JF779085 JF779457 3444RwITca Italy, Caselette 52 R. Savolainen and K.Vepsäläinen

JF779086 JF779458 3445RwITca Italy, Caselette 52 R. Savolainen and K.Vepsäläinen

JF779087 JF779459 3446RwITca Italy, Caselette 52 R. Savolainen and K.Vepsäläinen

JF779088 JF779460 3447RwITca Italy, Caselette 54 R. Savolainen and K.Vepsäläinen

JF779089 JF779461 3449RqITca Italy, Caselette 52 R. Savolainen and K.Vepsäläinen

JF779090 JF779462 3450RqITca Italy, Caselette 52 R. Savolainen and K.Vepsäläinen

JF779091 JF779463 3451RwITca Italy, Caselette 53 R. Savolainen and K.Vepsäläinen

JF779099 JF779471 3466RqITng Italy, Gattinara 52 R. Savolainen and K.Vepsäläinen

JF779100 JF779472 3467RqITng Italy, Gattinara 53 R. Savolainen and K.Vepsäläinen

JF779101 JF779473 3470RwITgo Italy, Gozzano 58 R. Savolainen and K.Vepsäläinen

JF779102 JF779474 3471RwITgo Italy, Gozzano 59 R. Savolainen and K.Vepsäläinen

JF779103 JF779475 3472RwITgo Italy, Gozzano 60 R. Savolainen and K.Vepsäläinen

JF779104 JF779476 3473RqITgo Italy, Gozzano 53 R. Savolainen and K.Vepsäläinen

JF779105 JF779477 3476RwITgo Italy, Gozzano 53 R. Savolainen and K.Vepsäläinen

JF779106 JF779478 3477RqITgo Italy, Gozzano 53 R. Savolainen and K.Vepsäläinen

JF779109 JF779481 3482RqITin Italy, Invorio 53 R. Savolainen and K.Vepsäläinen

JF779110 JF779482 3483RqITin Italy, Invorio 62 R. Savolainen and K.Vepsäläinen

JF779097 JF779469 3460RwITla Italy, Lenta 56 R. Savolainen and K.Vepsäläinen

JF779098 JF779470 3462RwITla Italy, Lenta 57 R. Savolainen and K.Vepsäläinen

JF779107 JF779479 3480RqITqu Italy, Quarna 53 R. Savolainen and K.Vepsäläinen

JF779108 JF779480 3481RwITqu Italy, Quarna 61 R. Savolainen and K.Vepsäläinen

JF779111 JF779483 3485RwITso Italy, Solbiate 8 R. Savolainen and K.Vepsäläinen

JF779112 JF779484 3486RwITso Italy, Solbiate 63 R. Savolainen and K.Vepsäläinen

JF779113 JF779485 3492RqITso Italy, Solbiate 60 R. Savolainen and K.Vepsäläinen

JF779114 JF779486 3496RqITso Italy, Solbiate 63 R. Savolainen and K.Vepsäläinen

JF779115 JF779487 3497RwITso Italy, Solbiate 63 R. Savolainen and K.Vepsäläinen

JF779116 JF779488 3498RwITso Italy, Solbiate 63 R. Savolainen and K.Vepsäläinen

JF779117 JF779489 3499RwITso Italy, Solbiate 53 R. Savolainen and K.Vepsäläinen

JF778996 JF779368 323RwKIR Kyrgyzstan, Issyk Kul 9 R. Schultz

JF779015 JF779387 3282RwNLut Netherlands, Utrecht 45 R. Savolainen and K.Vepsäläinen

JF779016 JF779388 3283RqNLut Netherlands, Utrecht 45 R. Savolainen and K.Vepsäläinen

JF779017 JF779389 3284RqNLut Netherlands, Utrecht 45 R. Savolainen and K.Vepsäläinen

JF779018 JF779390 3285RqNLut Netherlands, Utrecht 45 R. Savolainen and K.Vepsäläinen

JF779019 JF779391 3286RqNLut Netherlands, Utrecht 45 R. Savolainen and K.Vepsäläinen

JF779020 JF779392 3287RqNLut Netherlands, Utrecht 30 R. Savolainen and K.Vepsäläinen

JF779021 JF779393 3288RqNLut Netherlands, Utrecht 45 R. Savolainen and K.Vepsäläinen

JF779022 JF779394 3289RqNLut Netherlands, Utrecht 45 R. Savolainen and K.Vepsäläinen

JF779024 JF779396 3290RqNLut Netherlands, Utrecht 45 R. Savolainen and K.Vepsäläinen

JF779025 JF779397 3291RqNLut Netherlands, Utrecht 45 R. Savolainen and K.Vepsäläinen

JF779066 JF779438 337RwPLka Poland, Kampinos National Park 9 A. Stankiewicz

JF779070 JF779442 338RwPLka Poland, Kampinos National Park 9 A. Stankiewicz

JF779071 JF779443 339RwPLka Poland, Kampinos National Park 51 A. Stankiewicz

JF779073 JF779445 340RwPLka Poland, Kampinos National Park 9 A. Stankiewicz

JF779074 JF779446 341RwPLka Poland, Kampinos National Park 9 A. Stankiewicz

JF779215 JF779587 454RwPLwa Poland, Warsaw 14 A. Szczuka

JF779216 JF779588 455RwPLwa Poland, Warsaw 14 A. Szczuka

JF779217 JF779589 456RwPLwa Poland, Warsaw 14 A. Szczuka

JF779218 JF779590 457RwPLwa Poland, Warsaw 14 A. Szczuka

JF779219 JF779591 458RwPLwa Poland, Warsaw 14 A. Szczuka

JF779075 JF779447 342RwPLpu Poland, Wyszków 9 W. Czechowski

JF779080 JF779452 343RwPLpu Poland, Wyszków 13 W. Czechowski

JF778961 JF779333 3112RwROcl Romania, Cluj–napoca 36 B. Marco

JF778962 JF779334 3113RqROcl Romania, Cluj–napoca 3 B. Marco

JF778963 JF779335 3114RwROcl Romania, Cluj–napoca 3 B. Marco

JF778964 JF779336 3115RwROcmk Romania, Cluj–napoca 3 B. Marco

JF778957 JF779329 3108RwROst Romania, Saint Anne Lake 34 B. Marco

JF778958 JF779330 3109RwROst Romania, Saint Anne Lake 34 B. Marco

JF778959 JF779331 3110RwROst Romania, Saint Anne Lake 34 B. Marco

JF778986 JF779358 318RqROtu Romania, Turda 44 B. Marco

JF778960 JF779332 3111RwROva Romania, Valea Fagilor 35 B. Marco

JF778987 JF779359 319RwROvo Romania, Voslobeni 3 B. Marco

JF778988 JF779360 320RwROvo Romania, Voslobeni 3 B. Marco

JF778931 JF779303 3042RqRUac Russia, Akademgorodok 23 R. Savolainen and K.Vepsäläinen

JF778932 JF779304 3044RqRUac Russia, Akademgorodok 23 R. Savolainen and K.Vepsäläinen

JF778933 JF779305 3047mRUac Russia, Akademgorodok 24 R. Savolainen and K.Vepsäläinen

JF778934 JF779306 3047RwRUac Russia, Akademgorodok 25 R. Savolainen and K.Vepsäläinen

JF778935 JF779307 3049RqRUac Russia, Akademgorodok 13 R. Savolainen and K.Vepsäläinen

JF778939 JF779311 3053RqRUac Russia, Akademgorodok 13 R. Savolainen and K.Vepsäläinen

JF778940 JF779312 3054RqRUac Russia, Akademgorodok 13 R. Savolainen and K.Vepsäläinen

JF778941 JF779313 3056RqRUac Russia, Akademgorodok 13 R. Savolainen and K.Vepsäläinen

JF778942 JF779314 3059RqRUac Russia, Akademgorodok 25 R. Savolainen and K.Vepsäläinen

JF778944 JF779316 3061RqRUac Russia, Akademgorodok 8 R. Savolainen and K.Vepsäläinen

JF778947 JF779319 3068RwRUac Russia, Akademgorodok 30 R. Savolainen and K.Vepsäläinen

JF778948 JF779320 3074RwRUac Russia, Akademgorodok 13 R. Savolainen and K.Vepsäläinen

JF778945 JF779317 3065RqRUar Russia, Artybash 13 R. Savolainen and K.Vepsäläinen

JF778946 JF779318 3067RwRUar Russia, Artybash 29 R. Savolainen and K.Vepsäläinen

JF778980 JF779352 3129mRUbo Russia, Borisovka 42 A. Maisov

JF778981 JF779353 3129RwRUbo Russia, Borisovka 43 A. Maisov

JF778982 JF779354 3130RqRUbo Russia, Borisovka 43 A. Maisov

JF778983 JF779355 3131RqRUbo Russia, Borisovka 3 A. Maisov

JF778984 JF779356 3132RqRUbo Russia, Borisovka 43 A. Maisov

JF778985 JF779357 3133RqRUbo Russia, Borisovka 3 A. Maisov

JF778955 JF779327 3092RqRUbu Russia, Bugotak 3 R. Savolainen and K.Vepsäläinen

JF778956 JF779328 3097RwRUgo Russia, Gornyy 33 R. Savolainen and K.Vepsäläinen

JF778970 JF779342 3119RqRUlu Russia, Luvenga 9 A. Maisov

JF778971 JF779343 3120RqRUlu Russia, Luvenga 9 A. Maisov

JF778972 JF779344 3121RqRUlu Russia, Luvenga 9 A. Maisov

JF778973 JF779345 3122RqRUlu Russia, Luvenga 9 A. Maisov

JF778974 JF779346 3123RqRUlu Russia, Luvenga 9 A. Maisov

JF778952 JF779324 3086mRUma Russia, Mayak 31 R. Savolainen and K.Vepsäläinen

JF778953 JF779325 3087RqRUma Russia, Mayak 32 R. Savolainen and K.Vepsäläinen

JF778954 JF779326 3089RwRUma Russia, Mayak 9 R. Savolainen and K.Vepsäläinen

JF778989 JF779361 3210RwRUmo Russia, Moscow 14 R. Savolainen and K.Vepsäläinen

JF778990 JF779362 3211RqRUmo Russia, Moscow 14 R. Savolainen and K.Vepsäläinen

JF778991 JF779363 3212RqRUmo Russia, Moscow 9 R. Savolainen and K.Vepsäläinen

JF778992 JF779364 3213RqRUmo Russia, Moscow 9 R. Savolainen and K.Vepsäläinen

JF778937 JF779309 3051mRUno Russia, Novosibirk 27 R. Savolainen and K.Vepsäläinen

JF778938 JF779310 3051RwRUno Russia, Novosibirk 28 R. Savolainen and K.Vepsäläinen

JF778949 JF779321 3077mRUno* Russia, Novosibirk 27 R. Savolainen and K.Vepsäläinen

JF778950 JF779322 3078RwRUno* Russia, Novosibirk 13 R. Savolainen and K.Vepsäläinen

JF778951 JF779323 3080RqRUno Russia, Novosibirk 13 R. Savolainen and K.Vepsäläinen

JF778975 JF779347 3124RqRUpe Russia, Peterhof 8 A. Maisov

JF778976 JF779348 3125RqRUpe Russia, Peterhof 41 A. Maisov

JF778977 JF779349 3126RqRUpe Russia, Peterhof 41 A. Maisov

JF778978 JF779350 3127RwRUpe Russia, Peterhof 41 A. Maisov

JF778979 JF779351 3128RwRUpe Russia, Peterhof 41 A. Maisov

JF778943 JF779315 305RwSL Slovenia, Ptuj 8 R. Schultz

JF779188 JF779560 382_00RwSP Spain, Castiello de Jaca 1 R. Savolainen and K.Vepsäläinen

JF779190 JF779562 383_00RwSP Spain, Castiello de Jaca 1 R. Savolainen and K.Vepsäläinen

JF778923 JF779295 3001RqSEbr Sweden, Bromölla 18 R. Savolainen and K.Vepsäläinen

JF778922 JF779294 3000RwSEhu Sweden, Huaröd 17 R. Savolainen and K.Vepsäläinen

JF778927 JF779299 3023mSEjo Sweden, Jönåker 21 R. Savolainen and K.Vepsäläinen

JF778928 JF779300 3023RqSEjo Sweden, Jönåker 22 R. Savolainen and K.Vepsäläinen

JF779033 JF779405 32RqSEka Sweden, Karlshamn 45 R. Savolainen and K.Vepsäläinen

JF779072 JF779444 33RqSEka Sweden, Karlshamn 45 J. Leppänen

JF778924 JF779296 3010mSEkl* Sweden, Klavreström 19 R. Savolainen and K.Vepsäläinen

JF778925 JF779297 3011RwSEkl* Sweden, Klavreström 19 R. Savolainen and K.Vepsäläinen

JF778889 JF779261 25RwSEkr Sweden, Krankesjön 5 J. Leppänen

JF778890 JF779262 26RqSEkr Sweden, Krankesjön 6 J. Leppänen

JF778891 JF779263 27RqSEkr Sweden, Krankesjön 6 J. Leppänen

JF778920 JF779292 2999RqSEkr Sweden, Krankesjön 6 R. Savolainen and K.Vepsäläinen

JF778921 JF779293 29RwSEkr Sweden, Krankesjön 6 J. Leppänen

JF778930 JF779302 303RwCHbu Switzerland, Burtigny 1 Museum of Zoology of Lausanne

JF778936 JF779308 304RwCHbu Switzerland, Burtigny 26 Museum of Zoology of Lausanne

JF778929 JF779301 302RwCHch Switzerland, Cheserex 20 Museum of Zoology of Lausanne

JF778926 JF779298 301RwCHge Switzerland, Genolier 20 Museum of Zoology of Lausanne

JF779125 JF779497 3513RwCHje Switzerland, Jenins 66 R. Savolainen and K.Vepsäläinen

JF779126 JF779498 3514RwCHje Switzerland, Jenins 66 R. Savolainen and K.Vepsäläinen

JF779127 JF779499 3515RqCHje Switzerland, Jenins 66 R. Savolainen and K.Vepsäläinen

JF779128 JF779500 3516RqCHje Switzerland, Jenins 66 R. Savolainen and K.Vepsäläinen

JF779129 JF779501 3518RwCHje Switzerland, Jenins 66 R. Savolainen and K.Vepsäläinen

JF779130 JF779502 3519RqCHje Switzerland, Jenins 66 R. Savolainen and K.Vepsäläinen

JF779132 JF779504 3520RwCHje Switzerland, Jenins 67 R. Savolainen and K.Vepsäläinen

JF779133 JF779505 3521RqCHje Switzerland, Jenins 67 R. Savolainen and K.Vepsäläinen

JF779118 JF779490 3502RwCHpi Switzerland, Pian San Giacomo 64 R. Savolainen and K.Vepsäläinen

JF779119 JF779491 3505RwCHpi Switzerland, Pian San Giacomo 53 R. Savolainen and K.Vepsäläinen

JF779120 JF779492 3506RwCHpi* Switzerland, Pian San Giacomo 65 R. Savolainen and K.Vepsäläinen

JF779121 JF779493 3507mCHpi* Switzerland, Pian San Giacomo 65 R. Savolainen and K.Vepsäläinen

JF779122 JF779494 3510RwCHsa Switzerland, San Bernardino 53 R. Savolainen and K.Vepsäläinen

JF779123 JF779495 3511RqCHsa Switzerland, San Bernardino 53 R. Savolainen and K.Vepsäläinen

JF779124 JF779496 3512RqCHsa Switzerland, San Bernardino 53 R. Savolainen and K.Vepsäläinen

JF778913 JF779285 2892RwUAgo Ukraine, Golaya Pristan 9 R. Savolainen and K.Vepsäläinen

JF778914 JF779286 2894RwUAgo Ukraine, Golaya Pristan 9 R. Savolainen and K.Vepsäläinen

JF778915 JF779287 2912RqUAka Ukraine, Kaniv 9 R. Savolainen and K.Vepsäläinen

JF778916 JF779288 2921RqUAka Ukraine, Kaniv 8 R. Savolainen and K.Vepsäläinen

JF778917 JF779289 2924RwUAka Ukraine, Kaniv 9 R. Savolainen and K.Vepsäläinen

JF778894 JF779266 2824RwUAki Ukraine, Kiev 8 R. Savolainen and K.Vepsäläinen

JF778911 JF779283 2887mUAki Ukraine, Kiev 16 R. Savolainen and K.Vepsäläinen

JF778912 JF779284 2887RqUAki Ukraine, Kiev 8 R. Savolainen and K.Vepsäläinen

JF778909 JF779281 2885mUA* Ukraine, Luhyns'kyi 15 R. Savolainen and K.Vepsäläinen

JF778910 JF779282 2886RqUA* Ukraine, Luhyns'kyi 9 R. Savolainen and K.Vepsäläinen

JF778895 JF779267 2827RwUARwi Ukraine, Rivne 9 R. Savolainen and K.Vepsäläinen

JF778896 JF779268 2829RqUAvo1 Ukraine, Volin 10 R. Savolainen and K.Vepsäläinen

JF778897 JF779269 2833RqUAvo1 Ukraine, Volin 9 R. Savolainen and K.Vepsäläinen

JF778898 JF779270 2834RwUAvo1 Ukraine, Volin 9 R. Savolainen and K.Vepsäläinen

JF778899 JF779271 2841RqUAvo2 Ukraine, Volin 11 R. Savolainen and K.Vepsäläinen

JF778900 JF779272 2842mUAvo2* Ukraine, Volin 9 R. Savolainen and K.Vepsäläinen

JF778901 JF779273 2845RqUAvo2* Ukraine, Volin 9 R. Savolainen and K.Vepsäläinen

JF778902 JF779274 2849mUAvo2* Ukraine, Volin 12 R. Savolainen and K.Vepsäläinen

JF778903 JF779275 2850RqUAvo2* Ukraine, Volin 9 R. Savolainen and K.Vepsäläinen

JF778904 JF779276 2853RwUAvo3 Ukraine, Volin 13 R. Savolainen and K.Vepsäläinen

JF778905 JF779277 2871RqUAvo5 Ukraine, Volin 9 R. Savolainen and K.Vepsäläinen

JF778906 JF779278 2879RwUAvo6 Ukraine, Volin 14 R. Savolainen and K.Vepsäläinen

JF778907 JF779279 2882RqUAvo6 Ukraine, Volin 9 R. Savolainen and K.Vepsäläinen

JF778908 JF779280 2884RwUAvo4 Ukraine, Volin 9 R. Savolainen and K.Vepsäläinen

JF778993 JF779365 3216RwUSca USA, Cambridge 9 G. Alpert

JF778994 JF779366 3219RwUSca USA, Cambridge 45 G. Alpert

JF778892 JF779264 2822RwUSma USA, Maine 7 E. Groden

JF778893 JF779265 2823RwUSma USA, Maine 7 E. Groden

**Table S2.** List of haplotypes and their haplogroups of combined COI and Cyt b gene fragments and populations (capital letters refer to country, lowercase letters to area within country) where haplotype was detected, with numbers of individuals in parentheses.

**Haplotype Haplogroup Population (number of individuals)**

1 3-1 AUac (1), BEbr (3), CHbu (1), DKmo (1), ENdoki (8), ENdose (2), FRaz (4), FRla (5), FRmo (7), GEbb (1),

GEer (2), GEhe (6), GElb (2), GEri (6), GEst (2), SP (2)

2 3-1 FRmo (4)

3 3-6 BLvi (11), ROcl (2), Rocmk (1), ROvo (2), RUbo (2), RUbu (1)

4 3-6 BLvi (1)

5 3-1 SEkr (1)

6 3-1 SEkr (4)

7 3-1 USma (2)

8 3-3 FIpa (4), GEhe (6), FRmo (3), ITso (1), RUac (1), RUpe (1), SL (1), UAka (1), UAki (2)

9 3-2 FIhi (1), FIma (1), FIla (1), FIpa (2), FIot (1), FIpi (1), FIsi (2), FItu (2), FItv (6), FIuu (2), FIvi (5), KIR (1),

PLka (4), PLpu (1), RUlu (5), RUma (1), RUmo (2), UA (1), UAgo (2), UAka (2), UAri (1), UAvo1 (2), UAvo2 (3),

UAvo4 (1), UAvo5 (1), UAvo6 (1), USca (1)

10 3-3 UAvo1 (1)

11 3-2 UAvo2 (1)

12 3-7 UAvo2 (1)

13 3-4 PLpu (1), RUac (5), RUar (1), RUno (2), UAvo3 (1)

14 3-2 PLwa (5), RUmo (2), UAvo6 (1)

15 3-2 UA (1)

16 3-2 UAki (1)

17 3-1 SEhu (1)

18 3-7 SEbr (1)

19 3-7 SEkl (2)

20 3-1 CHch (1), CHge (1)

21 3-1 SEjo (1)

22 3-1 SEjo (1)

23 3-4 RUac (2)

24 3-2 RUac (1)

25 3-2 RUac (2)

26 3-1 CHbu (1)

27 3-4 RUno (2)

28 3-4 RUno (1)

29 3-2 RUar (1)

30 3-5 BEbr (1), NLut (1), RUac (1)

31 3-2 RUma (1)

32 3-6 RUma (1)

33 3-3 RUgo (1)

34 3-6 ROst (3)

35 3-2 ROva (1)

36 3-2 ROcl (1)

37 3-2 FIhi (1)

38 3-2 FIhi (1), FIve (1), FIpi (2)

39 3-2 FIva (1)

40 3-2 FIva (1)

41 3-2 RUpe (4)

42 3-3 RUbo (1)

43 3-3 RUbo (3)

44 3-3 ROtu (1)

45 3-7 AUac (3), BEbr (10), ENnoun (1), FRaz (2), GEbb (1), GEse (4), GEwi (7), NLut (9), SEka (2), USca (1)

46 3-1 FR (1)

47 3-3 AN (12)

48 3-7 BEbr (1)

49 3-3 GEhe (2)

50 3-7 AUac (1), GEbz (4), GEwi (8)

51 3-2 PLka (1)

52 3-1 ITng (1), ITca (12)

53 3-1 CHpi (1), CHsa (3), ITca (2), ITgo (3), ITin (1), ITng (1), ITqu (1), ITso (1)

54 3-1 ITca (1)

55 3-1 ITar (5)

56 3-1 ITla (1)

57 3-1 ITla (1)

58 3-1 ITgo (1)

59 3-1 ITgo (1)

60 3-3 ITgo (1), ITso (1)

61 3-1 ITqu (1)

62 3-1 ITin (1)

63 3-4 ITso (4)

64 3-3 CHpi (1)

65 3-3 CHpi (2)

66 3-7 CHje (6)

67 3-1 CHje (2)

68 3-3 GEer (1)

69 3-1 GEri (1)

70 3-7 GEnt (4), GEri (4)

71 3-7 GEri (2)

72 3-1 GEst (2)

73 3-7 GEst (2)

74 3-3 FRmove (1)

75 3-1 FRaz (1)

76 3-7 FRaz (3)

77 3-3 FIot (1)

78 3-4 FIsk (1)

79 3-1 ENnoun (2)

80 3-7 ENnoea (2)

81 3-7 ENdore (7)

82 3-1 ENdoki (1)

83 3-2 FIma (2)

84 3-2 FIpa (2)

85 3-3 FIpa (2)

86 3-2 FIsi (2)

87 3-2 FIsi (1)

88 3-2 FIve (1)

89 3-2 FIpi (2)

90 3-1 GElb (1)

91 3-2 GElb (1)

92 3-2 GElb (1)

93 3-7 GEbz (1)

94 3-7 GEbb (1)

95 3-2 FItv (2)

96 3-2 FItv (1)

97 3-2 FIvi (5)

98 3-2 FIvi (4)

99 3-3 FIvi (2)

100 3-2 FIvi (2)

101 3-3 FIvi (1)

**Table S3.** Genetic diversity estimates for host and parasite populations. Population (capital letters refer to country, lowercase letters to area within country), numbers of hosts and parasites in each population, haplotype diversity (*h* **±** standard deviation), genetic diversity (*π* **±** standard deviation) for host and parasite populations with more than one individual. Asterisk (*) indicates mean values for parasitised host populations.

**Population Individuals *h* ± SD π ± SD *h* ± SD π ± SD**

**Host Parasite Host Host Parasite Parasite**

AN 12 – 0.00 ± 0.00 0.0000 ± 0.0000 – –

AUac 5 – 0.70 ± 0.22 0.0025 ± 0.0018 – –

BEbr 15 – 0.54 ± 0.13 0.0039 ± 0.0022 – –

BLvi 12 – 0.17 ± 0.13 0.0001 ± 0.0002 – –

DKmo 1 – – – – –

ENdoki 9 – 0.22 ± 0.17 0.0002 ± 0.0002 – –

ENdore 7 – 0.00 ± 0.00 0.0000 ± 0.0000 – –

ENdose 1 1 – – – –

ENnoea 2 – 0.00 ± 0.00 0.0000 ± 0.0000 – –

ENnoun 3 – 0.67 ± 0.31 0.0042 ± 0.0034 – –

FIhi 2 1 1.00 ± 0.50 0.0028 ± 0.0031 – –

FIla 1 – – – – –

FIma 3 – 0.67 ± 0.31 0.0005 ± 0.0006 – –

FIot 2 – 1.00 ± 0.50 0.0014 ± 0.0017 – –

FIpa 8 2 0.71 ± 0.12 0.0006 ± 0.0005 0.00 ± 0.00 0.0000 ± 0.0000

FIpi 3 2 0.67 ± 0.31 0.0014 ± 0.0013 0.00 ± 0.00 0.0000 ± 0.0000

FIsi 3 2 0.67 ± 0.31 0.0009 ± 0.0010 0.00 ± 0.00 0.0000 ± 0.0000

FIsk 1 – – – – –

FItu 2 – 0.00 ± 0.00 0.0000 ± 0.0000 – –

FItv 7 2 0.48 ± 0.17 0.0010 ± 0.0008 1.00 ± 0.50 0.0007 ± 0.0010

FIuu 1 1 – – – –

FIVa 1 1 – – – –

FIve 1 1 – – – –

FIvi 8 11 0.75 ± 0.14 0.0023 ± 0.0015 0.64 ± 0.09 0.0020 ± 0.0013

FR 1 – – – – –

FRaz 10 – 0.78 ± 0.09 0.0036 ± 0.0021 – –

FRla 5 – 0.00 ± 0.00 0.0000 ± 0.0000 – –

FRmo 11 3 0.71 ± 0.08 0.0024 ± 0.0015 0.67 ± 0.31 0.0005 ± 0.0006

FRmove 1 – – – – –

GEbb 2 1 1.00 ± 0.50 0.0063 ± 0.0066 – –

GEbz 1 4 – – 0.00 ± 0.00 0.0000 ± 0.0000

GEer 3 – 0.67 ± 0.31 0.0037 ± 0.0031 – –

GEhe 14 – 0.66 ± 0.07 0.0033 ± 0.0019 – –

GElb 1 4 – – 1.00 ± 0.18 0.0050 ± 0.0036

GEnt 4 – 0.00 ± 0.00 0.0000 ± 0.0000 – –

GEri 13 – 0.72 ± 0.09 0.0036 ± 0.0021 – –

GEse 4 – 0.00 ± 0.00 0.0000 ± 0.0000 – –

GEst 6 – 0.80 ± 0.12 0.0037 ± 0.0024 – –

GEwi 15 – 0.53 ± 0.05 0.0004 ± 0.0004 – –

ITar 5 – 0.00 ± 0.00 0.0000 ± 0.0000 – –

ITca 15 – 0.36 ± 0.14 0.0003 ± 0.0003 – –

ITgo 6 – 0.80 ± 0.17 0.0028 ± 0.0019 – –

ITin 2 – 1.00 ± 0.50 0.0007 ± 0.0010 – –

ITla 2 – 1.00 ± 0.50 0.0014 ± 0.0017 – –

ITng 2 – 1.00 ± 0.50 0.0007 ± 0.0010 – –

ITqu 2 – 1.00 ± 0.50 0.0007 ± 0.0010 – –

ITso 7 – 0.71 ± 0.18 0.0021 ± 0.0014 – –

KIR 1 – – – – –

NLut 10 – 0.20 ± 0.15 0.0029 ± 0.0018 – –

PLka 5 – 0.40 ± 0.24 0.0006 ± 0.0006 – –

PLpu 2 – 1.00 ± 0.50 0.0014 ± 0.0017 – –

PLwa 5 – 0.00 ± 0.00 0.0000 ± 0.0000 – –

ROcl 3 – 0.67 ± 0.31 0.0051 ± 0.0041 – –

ROcmk 1 – – – – –

ROst 3 – 0.00 ± 0.00 0.0000 ± 0.0000 – –

ROtu 1 – – – – –

ROva 1 – – – – –

ROvo 2 – 0.00 ± 0.00 0.0000 ± 0.0000 – –

RUac 11 1 0.78 ± 0.11 0.0038 ± 0.0022 – –

RUar 2 – 1.00 ± 0.50 0.0021 ± 0.0024 – –

RUbo 5 1 0.60 ± 0.17 0.0042 ± 0.0028 – –

RUbu 1 – – – – –

RUgo 1 – – – – –

RUlu 5 – 0.00 ± 0.00 0.0000 ± 0.0000 – –

RUma 2 1 1.00 ± 0.50 0.0070 ± 0.0073 – –

RUmo 4 – 0.67 ± 0.20 0.0005 ± 0.0005 – –

RUno 3 2 0.67 ± 0.31 0.0009 ± 0.0010 0.00 ± 0.00 0.0000 ± 0.0000

RUpe 5 – 0.40 ± 0.24 0.0006 ± 0.0006 – –

SEbr 1 – – – – –

SEhu 1 – – – – –

SEjo 1 1 – – – –

SEka 2 – 0.00 ± 0.00 0.0000 ± 0.0000 – –

SEkl 1 1 – – – –

SEkr 5 – 0.40 ± 0.24 0.0008 ± 0.0007 – –

SL 1 – – – – –

SP 2 – 0.00 ± 0.00 0.0000 ± 0.0000 – –

CHbu 2 – 1.00 ± 0.50 0.0021 ± 0.0024 – –

CHch 1 – – – – –

CHge 1 – – – – –

CHje 8 – 0.43 ± 0.17 0.0027 ± 0.0017 – –

CHpi 3 1 1.00 ± 0.27 0.0051 ± 0.0041 – –

CHsa 3 – 0.00 ± 0.00 0.0000 ± 0.0000 – –

UA 1 1 – – – –

UAgo 2 – 0.00 ± 0.00 0.0000 ± 0.0000 – –

UAka 3 – 0.67 ± 0.31 0.0005 ± 0.0006 – –

UAki 2 1 0.00 ± 0.00 0.0000 ± 0.0000 – –

UAri 1 – – – – –

UAvo1 3 – 0.67 ± 0.31 0.0014 ± 0.0013 – –

UAvo2 3 2 0.67 ± 0.31 0.0005 ± 0.0006 1.00 ± 0.50 0.0021 ± 0.0024

UAvo3 1 – – – – –

UAvo4 1 – – – – –

UAvo5 1 – – – – –

UAvo6 2 – 1.00 ± 0.50 0.0007 ± 0.0010 – –

USca 2 – 1.00 ± 0.50 0.0014 ± 0.0017 – –

USma 2 – 0.00 ± 0.00 0.0000 ± 0.0000 – –

**Total** 371 48

Mean ± SD 0.52 ± 0.38 0.0015 ± 0.0017 0.43 ± 0.47 0.0010 ± 0.0016

0.71 ± 0.26* 0.0026 ± 0.0023*
